# Flavonoids from the Genus *Euphorbia:* Isolation, Structure, Pharmacological Activities and Structure–Activity Relationships

**DOI:** 10.3390/ph14050428

**Published:** 2021-05-02

**Authors:** Douglas Kemboi Magozwi, Mmabatho Dinala, Nthabiseng Mokwana, Xavier Siwe-Noundou, Rui W. M. Krause, Molahlehi Sonopo, Lyndy J. McGaw, Wilma A. Augustyn, Vuyelwa Jacqueline Tembu

**Affiliations:** 1 Department of Chemistry, Tshwane University of Technology, Pretoria 0001, South Africa; dinalamabatho@gmail.com (M.D.); nthabi.mokwana@gmail.com (N.M.); AugustynW@tut.ac.za (W.A.A.); 2 Department of Chemistry, Rhodes University, Grahamstown 6140, South Africa; r.krause@ru.ac.za; 3 Radiochemistry, South African Nuclear Energy Corporation, Pelindaba, Brits R104, South Africa; sonopom@yahoo.com; 4 Phytomedicine Programme, Department of Paraclinical Sciences, University of Pretoria, Private Bag X04 Onderstepoort 0110, Pretoria 0001, South Africa; lyndy.mcgaw@up.ac.za

**Keywords:** *Euphorbia*, flavonoids, pharmacological activities, structure–activity relationship

## Abstract

Plants of the genus *Euphorbia* are widely distributed across temperate, tropical and subtropical regions of South America, Asia and Africa with established Ayurvedic, Chinese and Malay ethnomedical records. The present review reports the isolation, occurrence, phytochemistry, biological properties, therapeutic potential and structure–activity relationship of *Euphorbia* flavonoids for the period covering 2000–2020, while identifying potential areas for future studies aimed at development of new therapeutic agents from these plants. The findings suggest that the extracts and isolated flavonoids possess anticancer, antiproliferative, antimalarial, antibacterial, anti-venom, anti-inflammatory, anti-hepatitis and antioxidant properties and have different mechanisms of action against cancer cells. Of the investigated species, over 80 different types of flavonoids have been isolated to date. Most of the isolated flavonoids were flavonols and comprised simple *O*-substitution patterns, C-methylation and prenylation. Others had a glycoside, glycosidic linkages and a carbohydrate attached at either C-3 or C-7, and were designated as d-glucose, l-rhamnose or glucorhamnose. The structure–activity relationship studies showed that methylation of the hydroxyl groups on C-3 or C-7 reduces the activities while glycosylation loses the activity and that the parent skeletal structure is essential in retaining the activity. These constituents can therefore offer potential alternative scaffolds towards development of new *Euphorbia*-based therapeutic agents.

## 1. Introduction

*Euphorbia* species are used in traditional medicine for the treatment of various diseases. Plants of the *Euphorbia* genus are common herbs that are applied in the treatment of respiratory diseases, healing of wounds, relieving skin irritations, indigestion, inflammation, microbial infestations and also as a food source [1,2,3,4]. Prehistorical records show that *Euphorbia* species were used in the treatment of scorpion and snake bites, liver diseases, respiratory disorders, asthma and rheumatism in the Chinese and Ayurveda medicine systems [3,4].

The medicinal applications of these species have been attributed to the presence of diverse secondary metabolites such as flavonoids and terpenes [5,6,7]. The abundance of these chemical constituents in *Euphorbia* species qualifies them as a rich source of therapeutic natural products possessing various pharmacological activities. These constituents can provide potential lead molecules for drug discovery. The genus *Euphorbia* is also among the largest of genera in the *spurge* family, and consists of several other subsections and subgenera, having more than 2000 species [8,9] with promising research potential.

Flavonoids are among the dominant constituents of *Euphorbia* species after macrocyclic diterpenes and triterpenoids [4,10]. Flavonoids mainly occur as isoflavonoids (3-phenylbenzopyrans), neoflavonoids (4-phenylbenzopyrans), chalcones [7,11], flavonols, flavanone, flavanonol, flavanol and anthocyanidins [11]. These constituents are structurally and biogenetically related as they share a common precursor, the chalcone [11,12]. They have also been reported to possess various pharmacological activities [12,13,14] and have promising therapeutic potential.

Apart from their biological functions in protecting plant species against herbivores and other pathogens as well as acting as stress-protecting agents, they also perform important pharmacological activities in humans. Reports indicate that plant flavonoids exhibit anti-ulcer, antidepressant, antimicrobial, antiviral, antibacterial, anti-diabetic, anti-inflammatory, anti-agiogenic [15], antiproliferative [16,17,18,19,20,21,22] and anticancer [6] activities in vitro. Even though they have not been classified as nutrients, the intake of flavonoids is considered to be significant for human health [23]. They are also used as natural dyes as well as for cosmetics and skin-care products [16,24]. Related studies have shown that flavonoids from the *Euphorbia* species also have a wide range of pharmacological activities such as cytotoxic, anti-inflammatory properties and tumor-promoting abilities [25,26].

Furthermore, various reports have stated the significance of flavonoids in metabolism of the thyroid hormone, which is commonly reported to be vitamin P and is considered useful in counteracting hemorrhage [27]. They are also functional foods for promotion of good health and prevention of diseases [27]. As a result, significant efforts have been made in isolation, identification and characterization of flavonoids from the latex, aerial parts, roots, stems, seeds, stem bark and whole plant extracts of some *Euphorbia* species since early times [28,29]. Indeed, several reviews have been published about the role and significance of plant flavonoids as a source of bioactive compounds. For instance, in 2019, Avtar and Bhawna [20] reviewed the chemistry and pharmacology of flavonoids, while Dieter [30] reported the significance of flavonoids in plant resistance to microbial attack.

Similarly, Muhammad et al. [7] reviewed the significance of flavonoids as prospective neuroprotectants in ageing associated with neurological disorders, while Ali et al. [31] reported the therapeutic role of flavonoids in bowel diseases. In addition, the role of plant flavonoids in cancer and apoptosis mechanisms was also reported [32], as well as the commercial application of flavonoids as anti-infective agents [14]. Efforts have also been made to review the occurrence of flavonoids in medicinal plants. For example, Bathelemy et al. [33] reported the occurrence, classification and biological activities of flavonoids from African medicinal plants, while Panche et al. [34] and Shashank and Abhay [35] reported the occurrence of flavonoids in selected species of different plant families. In addition, Nigel and Renee [36] reviewed over 796 naturally occurring flavonols, dihydroflavonols, chalcones, dihydrochalcones, aurones and anthocyanins.

However, even though other reports have reviewed the phytochemical constituents of *Euphorbia* species, most of the published reviews have exclusively focused on ethnomedicinal uses [2,3,4,5,6], isolated diterpenes [10], essential oils and triterpenoids [4]. For example, Goel et al. [37] reviewed the structural diversity of phorbol esters while Shi et al. [38] reported the pharmacological activities and chemical constituents of *Euphorbia* species. In addition, Andrea and co-authors reviewed the structural diversity of *Euphorbia* diterpenes covering the period 2008–2012, and their pharmacological activities [10], while Kemboi et al. [4] reported the ethnomedicinal uses and the structural diversity of *Euphorbia* triterpenoids. Rojas et al. [39] reviewed the phytochemical and functional properties of *E*. antisyphilitica Zucc, while Yang et al. [40] described the traditional uses, phytochemistry and pharmacological aspects of *E. ebracteolata* Hayata. In general, reports on *Euphorbia* species have exclusively focused on isolated diterpenes and triterpenes with limited reference to flavonoids, and other phenolic constituents such as flavonoids over the past two decades, and there is no report of isolated *Euphorbia* flavonoids within this period. Hence, in order to gain a more comprehensive understanding of *Euphorbia* flavonoids, the current review reports the occurrence, isolation, structure, pharmacological activities and the therapeutic potential of flavonoids of the genus *Euphorbia* covering the period 2000–2020. Harnessing this information can provide an updated database of compounds of the *Euphorbia* species that may provide potential hits for drug discovery or developing a useful pharmacopeia, as well as assisting in explaining the observed synergistic effect of the crude extracts.

## 2. Literature Sources and Search Strategy

Information about the *Euphorbia* flavonoids, their chemistry, biosynthesis, structure, biological activities, structure–activity relationships and potential therapeutic value was obtained through an online literature search using terms such as ‘*Euphorbia* flavonoids’, ‘*Euphorbia* constituents’, ‘biological activities of *Euphorbia* flavonoids’, ‘therapeutic potential’ and ‘structure–activity relationship of *Euphorbia* flavonoids’ using online databases such as Scopus, Scifinder, Wiley online, Springer Link, Science Direct, PubMed, and Google Scholar. The online search was customized between the years 2000 and 2020 which resulted into over 400 reports about *Euphorbia*, mainly in the English language that were easily accessible. Of these over 100 reports relevant to the study that described studies on isolation and elucidation of known and novel *Euphorbia* flavonoids, their biological activities, therapeutic potential and the structure–activity relationships were selected. The retrieved information was critically analyzed and searched for descriptions of previously described *Euphorbia* flavonoids, their occurrence (extraction solvent and plant part used), structures, their biological activities, therapeutic potential, and structure–activity relationships. Additional information was obtained by reviewing and analyzing the cited references in the selected articles. Hence, the present review provide an account of the previous and the latest information on *Euphorbia* flavonoids isolated between January 2000 and December 2020, their pharmacological activities, therapeutic potential and structure–activity relationships.

## 3. Flavonoids

**Figure 1:**
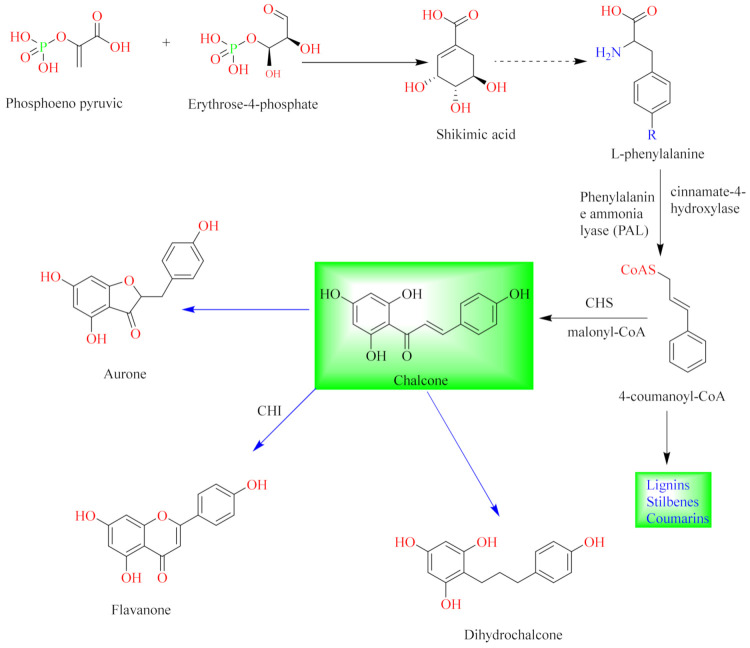
General biosynthetic pathway of flavonoids.

Flavonoids are natural compounds with various phenolic structures biosynthesized by plants [11]. Since the first isolation of a plant flavonoid, rutin, in 1930, there have been over 6000 different types of flavonoids identified from plant species to date [11,41]. The basic skeletal structure of flavonoids has 15 carbon atoms that are arranged to form a C_6_-C_3_-C_6_ ring system. They are divided into different classes based on their molecular structures and the degree of oxidation and unsaturation of the linking chain at C-3. They are biosynthesized in plants via the shikimic acid pathway [11,12,16] as summarized in Figure 1.

The initial stage is the condensation of a *p*-coumaroyl-CoA molecule with 3 molecules of malonyl-CoA to give chalcone in the presence of the chalcone isomerase enzyme. Chalcone is further isomerized by chalcone flavanone isomerase enzyme (CHI) to form flavanone. Thereafter, the pathway diverges to several other branches to produce different classes of flavonoids such as flavonols, flavones, flavonones or their dihydroderivatives [11,12] as illustrated in Figure 1.

**Figure 2:**
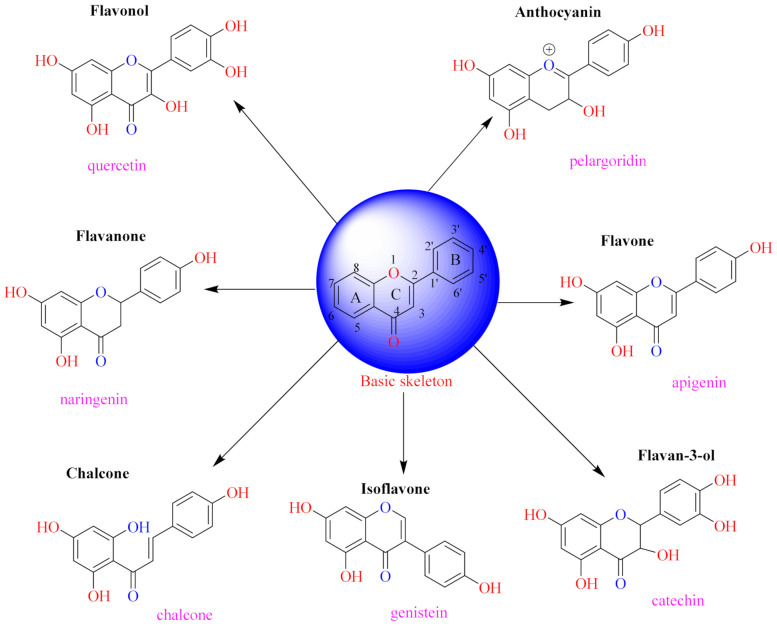
Representative structures of major classes of flavonoids.

The position of the benzenoid substituent is the basis of their classification into 2-phenylbenzopyrans, isoflavonoids (3-phenylbenzopyrans), neoflavonoids (4-phenylbenzopyrans) and chalcones. However, flavonols differ from flavonones by the hydroxyl substituent at C-3′ and C-2-C-3 double bonds (Havsteen, 1983). In most cases, flavonoids have the hydroxyl group at C-3, C-5, C-7, C-2′, C-3′, C- C-4′, C-5′. For flavonoid glycosides, the glycosidic linkage is unusually located at C-3 or C-7 and the carbohydrate attached can either be d-glucose, l-rhamnose, glucorhamnose, galactose or arabinose [42]. As a consequence, flavonoids are divided into seven major groups [35,43] as in Figure 2. However, further information on classification and biosynthesis of plant flavonoids is not dealt with in this review as the references can be consulted for detailed information.

## 4. Isolation of *Euphorbia* Flavonoids

Generally, flavonoids of the *Euphorbia* species are isolated using similar procedures as employed for other chemical constituents. Since all *Euphorbia* parts accumulate these constituents, the stems, aerial parts, roots, fruits, seeds, flowers and in some cases the whole plant are usually investigated. The aerial part is commonly studied since it is known to contain different phenolic compounds, especially flavonoids [44,45,46]. However, isolation of these constituents is a complex procedure, because they occur in small quantities as complex mixtures of sugars with similar or related structural parent skeletal framework. Thus, their isolation and identification require the use of a multistep method. The common procedure involves three main stages, including plant preparation, extraction and fractionation of the crude extracts into discrete portions of similar Rf values, and isolation/purification. The sample preparation mostly involves maceration of shade-dried and powdered plant material with methanol or ethanol at room temperature for several days. The resulted filtrates are concentrated under reduced pressure to afford crude extracts. The organic extracts are then subjected to column chromatography with differing step-gradient solvent systems as eluents. Thereafter, the concentrated fractions are monitored by thin-layer chromatography (TLC) using various solvent systems. In cases where the species are rich with flavonoids, the TLC chromatograms of the fractions that have been eluted display intense yellow or brown spots when stained with p-anisaldehyde-sulphuric acid mixture [7].

In some cases, due to trace levels and the complexity of these constituents, they are identified using updated techniques with better resolution and sensitivity such as ultraperformance liquid chromatography coupled with quadrupole tandem time of flight mass spectrometry (UPLC-Q-TOF-MS) or the more traditional techniques such as high-performance liquid chromatography mass spectrometry (HPLC-MS) or vacuum liquid chromatography (VLC) and rotation planar chromatography (RPC) over silica gel using step gradient elution [44].

## 5. Flavonoids Isolated from *Euphorbia* Species

The current report documents the isolation, identification, structure, biological activities, structure–activity relationships and the therapeutic potential of various types of flavonoids from more than 30 *Euphorbia* species in the past two decades as summarized in Table 1. Of the species that have been investigated, over 80 different types of flavonoids (**1**–**85**) have been isolated from the aerial parts, roots, seeds and whole plant of these species. Over 50 of these compounds were isolated from the aerial and roots extracts, representing about 90% of all flavonoids reported. It could therefore be suggested that the concentration of these chemical constituents is in the aerial and roots parts. Many of these compounds were derived from the ethanol or methanol extracts of these plant parts. Among the investigated species, *E. lunulata* (number of isolated flavonoids (*n*) = 33) [26,47], *E. humifusa* (*n* = 11) [48], *E. hirta* (*n* = 10) [49] and *E. tirucalli* (*n* = 8) [50] were frequently investigated species, with *E. lunulata* [26,47] recording the highest number of isolated flavonoids (*n* = 33). In contrast, *E. mygdaloides* [51], *E. paralias* [52], *E. stenoclada* [53], *E. altotibetic* [51,54], *E. allepica* [51,55] and *E. magalanta* [51] were the least-investigated species, having the least number of isolated flavonoids (*n* = 1). Others include *E. helioscopia* (*n* = 7) [56], *E. lathyris* (*n* = 3) [57], *E. humifusa* [58], *E. ebracteolata* (*n* = 4) [59] and *E. lunulata* [60]. Future studies should therefore be directed on these species, as they remain a promising source of bioactive constituents.

Most of the isolated flavonoids were flavonols and comprise simple *O*-substitution patterns, C-methylation, prenylation, and adducts of quercetin (**55**) and kaempferol (**30**). Others have a glycoside, glycosidic linkages and a carbohydrate attached at either C-3 or C-7, and have been designated as d-glucose such as kaempferol 3-*O*-glucoside (**31**), L-rhamnose such as kaempferol-3-l-rhamnoside (**37**), or glucorhamnose such as kaempferol-3-*O*-*α*-rhamnoside-*O*-*β*-d-glucopyranoside (**39**). Of these, prenylated and glycosylated flavonols remain the most abundant classes of reported flavonoids in the review period, representing about 80% of all reported flavonoids in *Euphorbia* species to date. However, flavanones and chalcones have also been reported within the review period. For instance, 2′,4,4′-trihydroxychalcone (**9**) [56], has been reported from *E. helioscopia*. In addition, Laila [48] reported the isolation of 3,5,3′-trihydroxy-6,7-d-methoxy-4′ (7”-hydroxygeranyl-1”-ether) flavone (**10**) and 5,7,8,3′,4′-pentahydroxy-3-methoxyflavone (**19**) from the methanol extracts of *E. paralais* and *E. retusa*, respectively. Reported literature also shows that the flavone category makes up the second-largest group of flavonoids of the *Euphorbia* species, and includes apigenin (**21**), *O*-methylated flavones such as acacetin (**20**) from *E. bivonae* as well as flavonoid glycosides such as rutin (**81**) from *E. guyoniana*, kaempferol-3-*O*-*α*-dirhamnoside-*O*-*β*-d-glucopyranoside (**39**) from *E. bivonae* and kaempferol-3-rutinoside (**15**) from *E. larica*, among others. Most of the studied species contain one or many different classes of these flavonoids. Table 1 gives a summary of the isolated flavonoids, their structures, occurrence and their pharmacological effect.

Some flavonoids such as rhamnetin-3-α-arabinofuranoside (**80**) from *E. amygdaloides*, kaempferol-3-glucuronide (**36**) from *E. lathyris* [52,57] and quercetin-3-*O*-*β*-d-glucopyranosyl-(1-4)-*O*-*α*-l-rhamnopyranoside (**73**) from *E. drancunculoides* were identified for the first time in these species. Phytochemical investigation of *E. humifusa* ethanol extracts resulted in isolation of 13 flavone glucosides. Among them were the uncommonly isolated apigenin-7-*O*-(6′′-*O*-galloyl)-*β*-d-glucopyranoside (**22**), luteolin-7-*O*-*β*-d-glucopyranoside (**49**), luteolin-7-*O*-(6′′-*O*-trans-feruloyl)-*β*-d-glucopyranoside (**48**) as well as luteolin-7-*O*-(6′′-*O*-coumaroyl)-*β*-d-glucopyranoside (**47**). It was interesting to note that luteolin-7-*O*-(6′′-*O*-trans-feruloyl)-*β*-d-glucopyranoside (**48**) and luteolin-7-*O*-(6′′-*O*-coumaroyl)-*β*-d-glucopyranoside (**47**) had similar features as apigenin-7-*O*-(6′′-*O*-galloyl)-β-d-glucopyranoside (**22**). The distinctive feature was that the parent structure of apigenin-7-*O*-(6′′-*O*-galloyl)-*β*-d-glucopyranoside (**22**) was apigenin (**21**) with a galloyl substitution on glucoside, while luteolin-7-*O*-(6′′-*O*-trans-feruloyl)-*β*-d-glucopyranoside (**48**) and luteolin-7-*O*-(6′′-*O*-coumaroyl)-*β*-d-glucopyranoside (**47**) was luteolin (**46**) with a feruloyl and coumaroyl substituent on the parent ring system, respectively [58].

In addition, flavonoids named kaempferol-3-*O*-*β*-d-glucopyranosyl-(1→4)-*α*-l-rhamnopyranosyl-(1→6)-*β*-d-galactopyranoside (**33**) and quercetin 3-*O*-6′′-(3-hydroxyl-3-methylglutaryl)-*β*-d-glucopyranoside (**60**) from *E. ebracteolata* were reported for the first time by Xin et al. [59]. This showed the structural diversity of *Euphorbia* flavonoids. A limited number of chalcones have also been isolated from *Euphorbia* species. For instance, licochalcone A (**44**), 2′,4,4′-trihydroxychalcone (**9**), licochalcone B (**45**), and glabrone (**25**) were isolated from *E. helioscopia* [56]. Most of these flavonoids were identified from the aerial extracts, which also reported significant pharmacological activities. Chemical investigation of the ethanolic root extracts of *E. tirucalli* using chromatographic procedures led to the isolation of a previously unreported flavonoid called myricetin (**5**) [50]. In addition, previously unreported quercetin 3-*O*-(2′,3′-digalloyl)-*β*-d-galactopyranoside (**56**) was isolated from the acetone whole plant extract of *E. lunulata* [60]. This compound showed weak antiproliferative activities and was found to mimic insulin that is bound with the galloyl group at the galactosyl moiety. Hence, it could become one of the seed molecules that can be used for the development of a nonpeptidyl insulin alternative medicine [60].

## 6. Biological Studies, Structure–Activity Relationship and Therapeutic Potential

### 6.1. Cytotoxic Studies

In vitro evaluation of the ethanolic extract of *E. stenoclada* for its antiproliferative activity against human airway smooth muscle cells (HASMC) was conducted by Chaabi et al. [53]. The results showed that the ethanolic extract abolished completely the interleukin-1*β* (IL-1*β*)-induced proliferation of HASMC with IC_50_ of 0.73 ± 0.08 µg/mL. However, there was reduced activity of the fractionated crude extracts, suggesting that the crude extract was active due to the synergetic effect of multiple compounds. In addition, no cytotoxic effects were exhibited up to 20 µg/mL. Quercetin (**55**), the major constituent isolated from this extract, showed moderate activity with IC_50_ of 0.49 ± 0.12 µg/mL.

The structure–activity relationship studies using methylated and glycosylated flavonols showed that methylation reduces the antiproliferative activities, while glycosylation lost the activity [53]. For all the quercetin heterosides used, none of them exhibited any activity on the 1*β* (IL-1*β*)-induced proliferation of HASMC, an indication that the presence of the hydroxyl group at C-3 is key in retaining the activity, as its substitution resulted in low activity or complete loss of it. In addition, methylation of any of the hydroxyl groups of flavonol also had a negative effect on the activity. This was the case even at higher concentrations, suggesting that the free hydroxyl groups of quercetin (**55**) are all important in retaining its activity and that its substitution by methylation and glycosylation results in a lowering or loss of its activity [53]. It was also found to have a relaxant effect on guinea pig trachea pre-contracted with histamine [78,87] as well as in vitro inhibition of histamine release from the rat peritoneal mast cells by 95–97% [88]. This shows the therapeutic potential of quercetin (**55**) as a promising antiasthmatic agent.

Analysis of the anticancer activities of the flavonoid rich extract of *E. lunulata* showed that it could inhibit the growth of Lewis lung cancer cells in mice and the rabbit serum. It was also shown to significantly inhibit the proliferation of lung cancer cells (A549) in a concentration–time dependent manner. For instance, at a concentration of 20% for 72 h, the proliferation rate of lung cancer cells was 39.08% [89]. The authors suggested that the plant extract could induce cell apoptosis by cell arrest in G1 phase. In addition, Gao et al. [90] found that the hexane extract of *E. lunulata* could inhibit the proliferation of human hepatoma (Hep-G2) cells also in a time–concentration dependent manner in vitro, with an inhibition rate of 0.063 at 2.5 µg/mL and 0.69 at 80 µg/mL after 48 h. This was further related to the mitochondrial pathways and/or cellular pathways of apoptosis.

The flavonoids eriodictyol (**82**) and naringenin (**83**) isolated from *E. metabelensis* tested negative on human normal cells (HeLa), breast cancer (MCF-7) and epithelial human breast cancer (MDA-MB-231) cell lines, and G-protein-gated inwardly rectifying potassium (GIRK) channel-blocking activities in vitro. The IC_50_ values for naringenin (**83**) were reported as 12.43 µM for HeLa, 5.78 µM for MCF-7, and 19.13 µM for MDA-MB-231 cells. Its blocking activity on GIRK channels was reported to be weak with inhibition of 12.18% ± 1.39 for eriodictyol (**82**) and 13.50% ± 2.69 for naringenin (**83**), at 10.00 µM. Despite the negative effect, these compounds possess promising anti-inflammatory, anti-allergenic, antimicrobial, and antioxidant properties [77,84,91,92,93].

Phytochemical analysis of 15 *Euphorbia* species revealed the presence of flavonoids from the methanol aerial extracts [51]. The plant extracts were evaluated for their in vitro anticancer properties against human liver cancer (HepG-2) and breast cancer (MCF-7) cell lines. The methanol extract of *E. lactea* exhibited good anticancer activities against HepG-2 and MCF-7 cell lines with IC_50_ of 5.20 and 5.10 µg/mL, respectively. Previous anticancer assays of ethanolic extracts from the same species displayed significant activities against a hepatic cancer cell line (HEp-2) with IC_50_ of 89.00 µg/mL [94]. In addition, similar studies have shown that the extract of *E. lactea* displayed anticancer and anti-migratory activities toward cellosaurus (HN22) cells [95]. It was also observed that the extracts of *E. officinarum* and *E. royleana* showed significant activities against human colon cancer adenocarcinoma cell lines (Caco-2) [51]. This was the first assay on the cytotoxicity of *E. officinarum* against these cancer cell lines. Methanol extracts of *E. trigona* reported moderate cytotoxicity against MCF-7 and Caco-2 cells, while previous studies on the latex of *E. trigona* against colon cancer cell lines (HT-29) were found to be inactive [96]. Notably, among the tested *Euphorbia* extracts, only *E. horrida*, *E. tirucalli* and *E. ingens* were inactive against all three tested cell lines. In contrast, previous studies reported good cytotoxic effects of *E. tirucalli* butanol extract against MCF-7 cells as well as against human leukocytes, with IC_50_ of between 100 and 150 µg/mL [97]. The observed anticancer activities were attributed to the presence of identified phenolic and flavonoid constituents in the plant extracts.

Chemical investigation of *E. paralias* whole-plant extracts afforded quercetin-3-*O*-*β*-d-glucoside (**62**). This compound exhibited moderate toxicity against human liver cancer (HepG-2) and human lung cancer (A549) cells with IC_50_ values of 41 and 36 µM, respectively [52]. It also displayed the ability to inhibit the glutamine synthase enzyme with IC_50_ of 40 µM. Due to the fact that this enzyme is a potential target in the development of new antimycobacterial agents and that it plays an important role as a virulent factor of *Mycobacterium tuberculosis*, quercetin-3-*O*-*β*-d-glucoside (**62**) could be suggested for development as a potential antituberculotic agent [52]. In addition, Salehi et al. [77] reported the antidiarrheal activities of quercitrin (**78**) isolated from *E. hirta* decoction in mice at doses of 50 mg/kg.

Cheng et al. [62] found that *E. helioscopia* had a high concentration of quercetin (**55**) flavonoid. Evaluation of the extracts showed that it effectively inhibited the growth of HepG2 (human hepatocellular carcinoma lines) at 50 μg/mL in vivo (*p* < 0.01).

Flavonoids show anti-inflammatory and antiproliferative properties through several mechanisms of action such as inhibition of protein kinase and transcription factors, inhibition of phosphodiesterase impact on arachidonic acid metabolisms and effects in immune system, among others. Since protein kinases are essential in signal transduction during cell activation in inflammation, some flavonoids are known to target multiple central kinases involved in the processes of multiple signaling pathways [98]. Flavonoids have also been reported to inhibit kinases such as protein kinase C, phosphatidylinositol kinase, phosphoinositol kinase, tyrosine kinase or cyclin-dependent kinase-4 [99,100]. They are suggested to modulate protein kinases by inhibiting the transcription factors, such as nuclear factor kappa-light chain enhancer of activated B cells (NF-κB) [101], which regulates different chemokines, cytokines and cell adhesion molecules that are involved in inflammation. For example, quercetin (**55**) has been used as an effective colorectal cancer agent and has been shown to exhibit different mechanisms of action, among them antioxidant activity, cell-cycle arrest, modulation of estrogen receptors, increase in apoptosis, inhibition of metastasis, regulation of signaling pathways and angiogenesis [102]. Luteolin (**46**) has shown anticancer activities in hepatocellular carcinoma (HCC) through a pro-apoptotic process and cell-cycle arrest at the G2/M stage [103].

In respect to the inhibition of phosphodiesterase enzyme impact on arachidonic acid metabolisms, flavonoids are known to inhibit the phosphodiesterases signals such as cAMP phosphodiesterase which is a key messenger molecule that regulates cell functions during inflammation stages. Flavonoids have the potential to block phosphodiesterases cAMP degradation and prolong cAMP signaling of the enzyme, thereby exhibiting anti-inflammatory properties [104]. Kaempferol (**30**) was found to stimulate body antioxidants against free radicals that may cause cancer [105], while myricetin (**5**) showed significant anti-inflammatory and anticancer activities by targeting different metabolic pathways in mitochondria that may result in cancer-cell death [106]. Furthermore, the synthetic polylactic-co-glycolic acid (PLGA) nanoparticles from the flavonoid hesperidin decreased the cell viability against C6 glioma cells [106,107]. In addition, the most common benzo-furanone, an anticancer flavonoid, was found to have different mechanisms of action against cancer cells as it has multiple targets such as acting on cyclin dependent kinase, adenosine receptor, histone deacetylase, microtubules, telomerase and sirtuins [108].

During inflammation, arachidonic acid is produced from the phospholipids of the plasma membranes by the phospholipase A2 (PLA2) enzyme. The acid is then oxidized by different oxygenases enzymes to produce thromboxanes, leukotrienes prostaglandins and other inflammatory mediators [109]. Flavonoids can inhibit such enzymes that are involved in this process (metabolism of arachidonic acid) and hence reduce the discharge of inflammatory mediators resulting from this pathway. Flavonoids are further suggested to inhibit the biosynthesis of thromboxanes, prostaglandins and leukotrienes by inhibition of the phospholipase A2 (PLA2) [110,111] and cyclooxygenase (COX) [112] enzymes.

Flavonoids can also inhibit maturation of dendritic cells (DCs) by suppressing maturation markers such as CD80, CD86, which are relevant for CD4+T lymphocytes cell activation [113,114]. Flavonoids influence the inflammatory response of dendritic cells by modulation of the iron metabolism [115]. Other studies have shown that some flavonoids decrease the discharge of histamine or prostaglandin from mast cells or can lead to inhibition of pro-inflammatory cytokines or chemokine production in neutrophils, mast cells and other immune cells [116,117,118]. It has also been demonstrated that flavonoids can bind to cytokine receptors such as the IL-17RA subunit of the IL-17 receptor, leading to the attenuation in its signaling. They can also inhibit the downstream signaling from receptors such as the high affinity receptor and other receptors at the site of inflammation [119].

### 6.2. Antioxidant Activities

Phytochemical investigation of *E. neriifolia* resulted in identification of the 2-(3,4-dihydroxy-5-methoxy-phenyl)-3,5-dihydroxy-6,7-dimethoxychromen-4-one (**8**) flavonoid from the leaf ethanol extract. This compound scavenged free radicals and reactive oxygen species (ROS), and inhibited lipid peroxidation with antioxidant activity of 76.15% compared to ascorbic acid at 75.6%. This suggests that compound (**8**) may have anticancer potential if such activity can be replicated in vivo. The high concentration of such flavonoids in *E. neriifolia* has been predicted to be responsible for the observed antioxidative mechanisms, which may be useful therapeutically, as oxidation has been implicated in causing several degenerative diseases [61].

A previous study by the same group reported that the pre-hepato-carcinogenesis, which is commonly induced by *N*-nitrosodiethylamine (DENA), was inhibited by the 70% hydro-ethanolic (*v*/*v*) extract of *E. neriifolia* and by the isolated flavonoid (**8**) [120]. These bioactive constituents, especially flavonoids and saponins, are known to neutralize the free radicals and other metabolic intermediate products that are highly reactive due to the presence of an unpaired electron. Hence, they are attributed to the observed protective histological effects. These findings could therefore be essential in validation of the ethnomedicinal uses and therapeutic potential of this plant.

Phytochemical investigation of *E. lathyris* showed that the root extract has the highest concentration of rutin flavonoids. However, the antioxidant evaluation of the testa, root and seed extracts showed that 2,2-diphenyl-1-picrylhydrazyl (DPPH) free-radical scavenging activity was highest for the testa extracts (61.29 ± 0.29 mmol Trolox/100 g dry weight of free compounds), while the highest ferric-reducing antioxidant power was shown by the seed extracts (1927.43 ± 52.13 mg FeSO_4_/100 g dry weight of free compounds). It was also established that the DPPH free-radical scavenging activities are dependent on the total phenolic content for different parts of *E. lathyris* extracts, which means that the higher the concentration of flavonoids and other phenolic compounds, the higher is the activity [121].

The postulated mechanism of action of these flavonoids as antioxidants is protection against lipid peroxidation that results in cell death. Quercetin (**55**), a free radical scavenger, was found to exert a protective effect. Furthermore, it was found to prevent free-radical-induced tissue damage in different ways. The most common is suggested to be the direct scavenging of free radicals. By doing so, the flavonoid, specifically quercetin (**55**), could inhibit the oxidized low-density lipoprotein (LDL) oxidation in vitro [122]. This action is known to protect against atherosclerosis. In addition, quercetin (**55**) can offer other potential therapeutic application in the prevention and treatment of allergies, fever and asthma. It was also found to work better when it is used in combination with bromelain [122]. Indeed, flavonoids such as quercetin (**55**) are common in foods such as apples, onions, tea, nuts, berries, cauliflower and cabbage among others. They can therefore provide many health-promoting benefits such as improving cardiovascular health, treatment of eye diseases, arthritis and, allergic disorders, as well as reducing the risk of cancers. Chemical analysis of the ethyl acetate aerial extracts of *E. geniculata* resulted in isolation of rutin (**81**), quercetin-3-*O*-rhamnoside (**75**), quercetin-3-*O*-β-D- glucopyranoside (**65**), and quercetin (**55**). The nephroprotective potential of the plant extract was further evaluated in male rats with thioacetamide-induced kidney injury. The results showed marked nephrotoxicity and were suggested to be through the alteration of kidney biomarkers and improving the redox status of the tissue, hence bringing the serum biochemical parameters to normal levels [123].

Within the larger *Euphorbiaceae* species, other genera have been reported to contain antioxidant flavonoids. For instance, chemical analysis of *Croton* species; *C. andinus*, *C. argentines*, *C. catamarcensis*, *C. cordobensis, C. curiosus, C. lachnostachyus, C. lanatus, C. saltensis* and *C. serratifolius* revealed the presence of *O*-glycosides flavonols, including kaempferol, quercetin and isorhamnetin as well as flavones such as apigenin. There was a significant degree (*p* > 0.05) of correlation between relative abundances of phenolic content and quercetin derivatives in these species, with the reducing antioxidant potential [124].

### 6.3. Anti-Hepatitis Activities

All the isolated flavonoids from *E. humifusa* were evaluated for their anti-HBV activity in vitro [58]. The compounds were assayed for their anti-HBV potential by observing the inhibitory secretion of hepatitis B surface antigen (HBsAg) and hepatitis B e-antigen (HBVe, HBeAg) in HBV-infected HepG-2 cells, at a non-cytotoxic concentration. The flavonoids apigenin-7-*O*-*β*-d-glucopyranoside (**23**) and apigenin-7-*O*-(6′′-*O*-galloyl)-*β*-d-glucopyranoside (**22**) significantly blocked the secretion of HBsAg and HBeAg in a dose-dependent manner. Apigenin-7-*O*-*β*-d-glucopyranoside (**23**) inhibited HBsAg secretion and HBeAg secretion by 77.2% and 55.5%, respectively, at a non-cytotoxic concentration of 40 μgmL^−1^, This was slightly higher than that of apigenin-7-*O*-(6′′-*O*-galloyl)-*β*-d-glucopyranoside (**23**) with inhibition of 82.2% and 65.6% for HBsAg and HBeAg secretion, respectively, at non-cytotoxic concentrations of 80 μgmL^−1^. Apigenin-7-*O*-(6′′-*O*-galloyl)-*β*-d-glucopyranoside (**22**) has a similar structure to apigenin-7-*O*-*β*-d-glucopyranoside (**23**) except for the substitution of a galloyl group on C-6 of glucoside. Therefore, it was postulated that the galloyl group could be important in retaining the anti-HBV activity [58].

**Figure 3:**
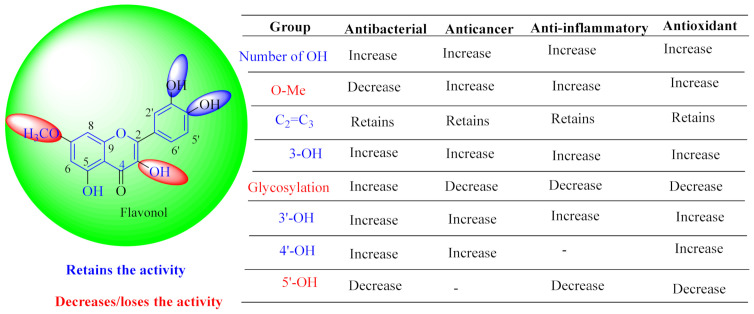
Summary of key pharmacopheric elements of flavonoids.

In contrast, luteolin-7-*O*-*β*-d-glucopyranoside (**49**) and luteolin-7-*O*-(6′′-*O*-trans-feruloyl)-β-D-glucopyranoside (**48**) showed weaker anti-HBV activity, as they had 50% and 33.9% secretion inhibition of HBsAg and HBeAg, respectively, at a non-cytotoxic concentration of 30 μgmL^−1^. In addition, luteolin-7-*O*-(6′′-*O*-coumaroyl)-*β*-d-glucopyranoside (**48**) displayed weak activity with secretion inhibition of HBsAg and HBeAg at 18.6% and 58.9% at a concentration of 80 μgmL^−1^. Luteolin (**46**), and quercetin (**55**), on the other hand, were inactive in relation to their high cytotoxicity. In addition apigenin-7-*O*-*β*-d-apiofuranosyl (1→2)-*β*-d-glucopyranoside (**85**), quercetin-3-*O*-α-L-rhamnosyl(1→6)-*β*-d-galactoside (**84**), quercetin-3-*O*-*β*-d-glucopyranoside (**65**) and quercetin-3-*O*-*β*-D-galactoside (**74**) (quercetin glucoside) showed no effect. The structure–activity relationship studies revealed that the parent structure (Figure 3**)** was essential to the anti-HBV activity of these compounds. It was also established that the number of glucosides in the parent structure could significantly influence their cytotoxicity. Furthermore, substitution of an acyl moiety for the glucoside is also important in retaining the anti-HBV activities of these compounds [58].

Analysis of the aerial part extracts of E. *microsciadia* resulted in isolation of four (**1**–**3**, **65**) flavonoids for the first time from this species [45]. These compounds were further evaluated for their immunomodulatory activities in vitro. The results showed lower lymphocyte suppression activity for all the flavonoids compared to prednisolone, the standard drug. It was also established that the suppressive activities of flavonoids having a hydroxyl group at both C-3′-and C-4′ in their B-ring such as quercetin 3-*O*-*β*-d-galactopyranoside (**3**) were more active than those with C-3′, C-4′ and C-5′ hydroxyl substitution as in myricetin 3-*O*-*β*-d-galactopyranoside (**2**). In addition, quercetin 3-*O*-*β*-d-rutinoside and myricetin 3-*O*-*β*-d-galactopyranoside (**2**) were inactive even at a higher concentration of 50 μg/mL [45]. It can therefore be rationalized that the hydroxyl groups at C-3, C-3′, C-4′, C-5′ and the parent structure are essential in retaining the activities and that glycosylation and methylation of these hydroxyl groups lowers the activity. Hence, they can be considered as key pharmacopheric elements of flavonoids as illustrated in Figure 3.

This was in agreement with previous reports that showed reduction of in vitro biological activities of the glycosylated form of these flavonoids [125]. Glycosylation of quercetin flavonoids reduces the in vitro biological activities compared to their corresponding aglycone forms. The lymphocyte antiproliferative activities of these compounds suggests that the type and the size of the sugar moiety influences the suppression activity on the T-cells. In other similar studies, using five different cell lines (colorectal adenocarcinoma (HT-29), lung carcinoma (A549), breast cancer (MCF-7), hepatocellular carcinoma (HepG-2) and colorectal carcinoma (HCT-116)), quercetin 3-*O*-*β*-d-galactopyranoside (**2**) showed the highest growth inhibitory rate of 20% in HT-29 and HepG-2, while rutin (**81**) recorded an inhibitory rate of 15% [126]. Flavonoids have also been suggested to have the capability to inhibit the production of superoxide enzymes such as xanthine oxidase and protein kinase C (PKC). PKC plays a significant role in the activation of T-cells. Hence, the inhibition of PKC by these compounds could be suggested to be their mechanism of action for the observed lymphocyte antiproliferative activities [126].

### 6.4. Anti-Venom Activities

Quercetin-3-*O*-rhamnoside (**72**) from the methanol extract of *E. hirta* demonstrated inhibition of the protease, phospholipase (PLA_2_), hyaluronidase and hemolytic activity of lyophilized snake venom. There was also an increase in survival time of mice that were injected with a mixture of snake venom with quercetin-3-*O*-rhamnoside (**72**). An increase in concentration of quercetin led to a reduction in edema to 107%, suggesting the inflammation inhibition that is caused by the venom. This could validate the medicinal application of this species traditionally in treating snake and scorpion poisoning [2,4]. Exploring such multifunctional plant molecules with anti-venom activities could further help in development of alternative and complementary medicine for snake- and scorpion-bite treatments, particularly in rural areas where the distribution of snake antivenom is not available. Furthermore, in silico molecular-docking analysis showed that quercetin-3-*O*-rhamnoside (**72**) interacts more efficiently through hydrogen bonds. The presence of a sugar substituent in quercetin-3-*O*-rhamnoside (**72**) was found to enhance the enzyme-ligand interactions. This was supported by findings of molecular dynamic simulations in vitro that showed the presence of various amino acid residues at the substrate binding sites of quercetin-3-*O*-rhamnoside (**72**) over quercetin (**55**). This study can therefore provide a scientific basis for the use of *E. hirta* extracts in traditional medicines [81]. In a related study, two *Jatropha* (*Euphorbiaceae*) species showed marked anti-edematogenic activities. While there was no observed difference when the extracts of the two species (*J. mollissima* and *J. gossypiifolia*) were administered by oral route (*p* > 0.05), through the intraperitoneal route *J. gossypiifolia* exhibited promising anti-edematogenic activity (*p* < 0.001) higher than *J. mollissima*. In antimicrobial studies, only *J. gossypiifolia* displayed antibacterial activity against *Staphylococcus aureus, Staphylococcus epidermidis* and *Bacillus cereus* with MIC value of 6.0 µg/µL compared to Vancomycin with MIC value of 0.5 µg/µL [127].

### 6.5. Antimalarial Activities

The methanol extract of *E. hirta* aerial parts exhibited 90% growth inhibition against *Plasmodium falciparum* at 5 µg/mL and displayed low toxicity against multidrug-resistant KB 3-1 cells [16]. This demonstrates the potential of this plant as an antimalarial agent, which supports the traditional uses in treatment of microbial infections [2]. Moreover, from this extract through a bio-guided methodology, flavonols were identified as quercitrin (**78**), and myricitrin (**53**). These flavonoids were able to inhibit the proliferation of the protozoan parasite responsible for malaria disease, *Plasmodium falciparum* strains FCR-3 (cycloguanil-resistant from Gambia) and CDC1 (chloroquine sensitive), with similar IC_50_ values 2.50 to 11.60 µM, respectively [16]. Antiplasmodial studies of ethanol extracts of the *E. hirta* whole plant showed that the flavonoid rich ethanol extracts did not suppress the chloroquine-sensitive strains of *Plasmodium* in vivo. The extract reduced the parasetemia levels at 44.36% compared to Camosunate (68.35%) [128]. Moreover, in vivo studies on the effects of flavonoids on mean arterial blood pressure and heart rate in albino rats showed that isoaromadendrin-7-*O*-*β*-d-glucopyranoside (isosinemsin) isolated from *E. cuneata* exhibited a decrease in blood pressure and heart rate at 16.6 mmHg and 16.6%, respectively [87].

### 6.6. Antibacterial and Antifungal Activities

Evaluation of extracts and isolated compounds from *E. tirucalli* displayed significant antibacterial and antifungal activities of the extracts against *Staphylococcus aureus* ATCC 6538, *S. brasiliensis* UFPE 121, *E. coli* ATCC 8739 and *C. albicans* UFPE 0231, with minimum inhibition (MIC) values ranging between 256 to 1024 μg/mL. Ampelopsin (**4**) was the most active compound with MIC value of 16 μg/mL against *E. coli* ATCC 8739, compared to tetracycline, with MIC value of 32 μg/mL. This demonstrates the antibacterial potential of *Euphorbia* flavonoids compared to conventional antibiotics. In antifungal studies, myricetin (**5**) showed the highest activity compared to amphotericin B against *C. albicans* UFPE 0231 with MIC value of 32 μg/mL compared to amphotericin B (16 μg/mL) [50]. Similarly, chemical profiling of the hexane extract of *E. royleana* revealed high phenolic and flavonoid contents and displayed significant antimicrobial activities [129]. The extract exhibited antifungal activity against *Aspergillus niger* and antibacterial activity against the Gram-positive bacteria *Bacillus subtilis* [129]. In addition, antibacterial evaluation of *E. guyoniana* extracts showed that the strains used were more sensitive to the flavonoids fractions of *E. guyoniana* with MICs varying from 1.47 to 61.78 mg/mL in the order of *Staphylococcus aureus* > *Streptococcus faecalis* > *Escherichia coli* [130].

In related studies, antibacterial activities of flavonoids from other genera in the *Euphorbiaceae* family have been reported. For instance, kaempferol 7-*O*-β-d-(6″-*O*-cumaroyl)-glucopyranoside, isolated from *Croton piauhiensis* (*Euphorbiaceae*) leaves, was evaluated for its antibacterial activities. The intrinsic antimicrobial activities and enhancement properties of this compound against *Pseudomonas aeruginosa Escherichia coli* and *Staphylococcus aureus* strains were further investigated. The results revealed that kaempferol 7-*O*-β-d-(6″-*O*-cumaroyl)-glucopyranoside had no antibacterial activity against strains tested at concentrations <1024 μg/mL. The combination of kaempferol 7-*O*-β-d-(6″-*O*-cumaroyl)-glucopyranoside at a concentration of 128 μg/mL with gentamicin exhibited synergistic effects against *S. aureus* and *E. coli* and also reduced the minimum inhibition (MIC) from 16 μg/mL to 4 μg/mL and 8 μg/mL, respectively [131]. In contrast, Ali et al. [132] showed that 5-7-dihydroxyflavone from *Oroxylum indicum* inhibited the growth of Gram-negative bacteria such as *E. coli* and *P. aeruginosa*. In the same study, the authors reported that the baicalein flavonoid exhibited weak activities against Gram-positive bacteria such as *Bacillus subtilis* and *S. aureus.* The weak activity was attributed to the presence of a new group at C-6. The activities with synergistic effects were attributed to the hydroxyl phenyl groups that have high affinity for proteins [133].

**Figure 4:**
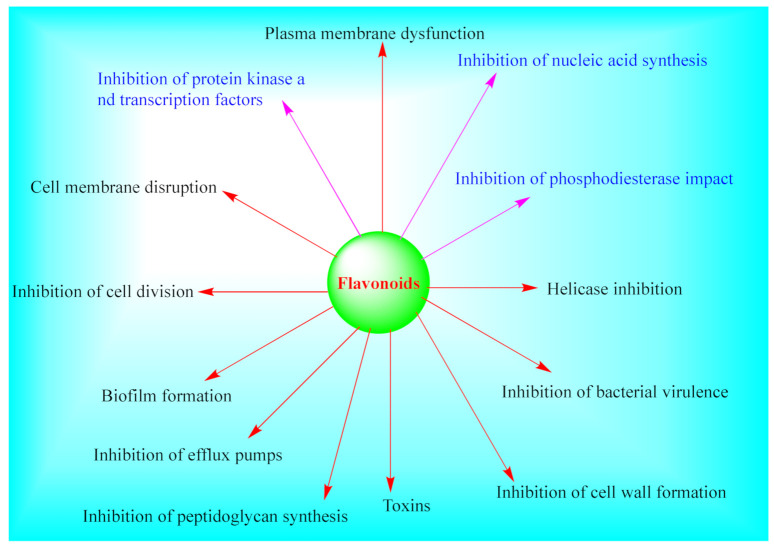
Summary of antibacterial and antifungal mechanisms of actions of flavonoids.

Even though the mechanisms of action of various *Euphorbia* flavonoids have not been fully explored, various mechanisms of action of plant flavonoids have previously been fronted. Flavones are suggested to form a complex with components of the cell wall and thus inhibit further adhesions or microbial growth. For instance, licoflavone C isolated from *Retama raetam* flowers was found to be active against *E. coli* via formation of complexes with extracellular and soluble proteins with MIC value of 7.81 μg/mL [134]. Other postulated mechanisms include inhibition of bacterial enzymes (such as tyrosyl-tRNA synthetase) [135], inhibition of bacterial efflux pump and rise in the susceptibility of existing antibiotics [136], change in cytoplasmic membrane function, nucleic acid synthesis inhibition, decrease in cell attachment, inhibition of energy metabolism, formation of biofilm, changing in membrane permeability, attenuation of the pathogenicity [137,138], damage of the cytoplasmic membrane [137,138], inhibition of nucleic acid synthesis (for instance, the inhibition of DNA gyrase from *E. coli* by quercetin (**55**), and apigenin (**21**) [139]) among others, as summarized in Figure 4. It was further established that the combination of apigenin and ceftazidime damages the cytoplasmic membrane of ceftazidime-resistant enterobacter cloacae, leading to subsequent leakage of intracellular components [140].

## 7. Conclusions and Future Perspectives

The extensive utilization of *Euphorbia* species in traditional and complementary medicines for treatment of various diseased conditions has led to increased interest in their phytochemistry and in vitro as well as in vivo studies using cells and animal models. The present review comprises a detailed phytopharmacological account of information available on *Euphorbia* flavonoids between 2000 and 2020. The findings suggest that the extracts and isolated flavonoids possess anticancer, antiproliferative, antimalarial, antibacterial, antivenom, anti-inflammatory, anti-hepatitis and antioxidant properties. Of these, antioxidant and anticancer activities are the most studied biological activities, partly due to the ethnomedicinal application of these species as anticancer agents. Indeed, it is widely accepted that the crude extracts have a synergetic effect compared to individual bioactive compounds. Nonetheless, only a handful of studies assessed the pharmacological potential of *Euphorbia* flavonoids as most studies employed the whole crude extracts. This limits the translational value as researchers are not equipped to determine whether the observed activities are related to the actions of a single bioactive compound or the synergy between multiple constituents present. It was also reported that these flavonoids possess different mechanisms of action against cancer cells. For instance, quercetin exhibited different mechanisms of action such as antioxidant activity, cell-cycle arrest and modulation of estrogen receptors among others. This is essential towards development of a potent anticancer agent. Of the investigated species, over 80 different types of flavonoids have been isolated from the aerial parts, roots, seeds and whole plant of these species. Most of the isolated flavonoids were flavonols and comprised simple *O*-substitution patterns, C-methylation and prenylation. Others had glycoside, glycosidic linkages and a carbohydrate attached at either C-3 or C-7, and were designated as d-glucose, l-rhamnose or glucorhamnose. A limited number of chalcones were also reported. The structure–activity relationship studies showed that methylation of hydroxyl groups at C-3 or C-7 reduces the activities, while glycosylation results in loss of activity. These constituents can therefore offer a potential alternative for development of therapeutic agents based on the *Euphorbia* species.

While the overall findings suggest a promising future with an abundance of therapeutic potential of the *Euphorbia* species, there are still many aspects of research on these species that need to be considered. These include using species from different ecological zones, adoption of high throughput screening strategies and metabolomics tools for discovery of new bioactive compounds in complex plant matrices, toxicological studies, detailed mechanistic studies and molecular analysis. Furthermore, the current evidence is largely limited to the unverified ethnomedicinal application of these species in folk medicine and their pharmacological studies in vitro. Essentially, more robust scientific studies are needed before confirmatory decisions can be made on the therapeutic potential of flavonoids from *Euphorbia* species.

## Figures and Tables

**Table 1:** Reported flavonoids from *Euphorbia* species.

No	Compound Structure and Name	Classification	Species Name	Plant Part, Solvent	Biological Effect	Reference
1.	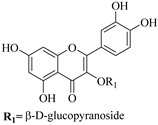 quercetin 3-*O*-*β*-d-rutinoside	Glucosidic flavonol	*E. microsciadia*	Aerial, EtOH	Antiproliferative	[45]
2.	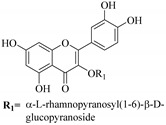 myricetin 3-*O*-*β*-d-galactopyranoside	Glucosidic flavonol	*E. microsciadia*	Aerial, EtOH	Antiproliferative	[45]
3.	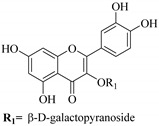 quercetin 3-*O*-*β*-d-galactopyranoside	Glucosidic flavonol	*E. microsciadia*, *E. heterophylla*	Aerial, EtOH	Antiproliferative	[45,46]
4.	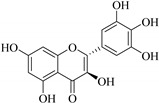 ampelopsin	Flavonol	*E. tirucalli*	Whole plant, EtOH	Antibacterial, antifungal	[50]
5.	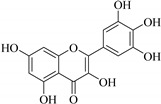 myricetin	Flavonol	*E. tirucalli*	Whole plant, EtOH	Antibacterial, antifungal	[49]
6.	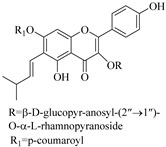 hirtacoumaroflavonoside (7-*O*-(p-coumaroyl)-5,7,4-trihydroxy-6-(3,3-dimethylallyl)-flavonol-3-*O*-*β*-d-glucopyr-anosyl-(2″→1″′)-*O*-*α*-l-rhamnopyranoside)	Carbohydrate flavonol	*E. hirta*	Roots, MeOH	Inhibitory activity (α-glucosidase)	[49]
7.	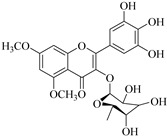 dimethoxyquercitrin	Glucosidic flavonol	*E. hirta*	Roots, MeOH	Inhibitory activity (α-glucosidase)	[49]
8.	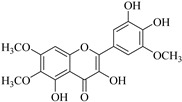 2-(3,4-dihydroxy-5-methoxy-phenyl)-3,5-dihydroxy-6,7-dimethoxychromen-4-one	Flavonol	*E. neriifolia*	EtOH, leaves	Ant-oxidant	[61]
9.	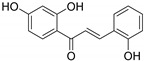 2′,4,4′-trihydroxychalcone	Chalcone	*E. helioscopia*	Whole plant, EtOH	Not evaluated	[56]
10.	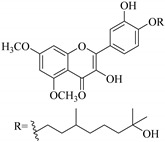 3,5,3′-trihydroxy-6,7-dimethoxy-4′ (7”-hydroxygeranyl-1”-ether) flavone	Flavone	*E. paralais*	Whole plant, MeoH	Not evaluated	[48]
11.	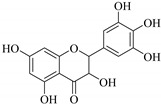 3,5,7-trihydroxi-2-(3′,4′,5′ trihidroxefenil)-2,3 dihidrobenzopiran-4-ona	Flavonol	*E. tirucalli*, *E. helioscopia*	Roots, EtOH	Antimicrobial	[50,56,62]
12.	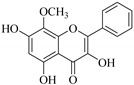 3,5,7-trihydroxy-8- methoxyflavone	Flavone	*E. lunulata*	Aerial, EtOH		[26,47]
13.	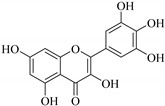 3,5,7-trihydroxy-2-2(3′, 4′, 5′-trihydroxyphenyl) benzopyran-4-one	Flavonol	*E. tirucalli*	Roots, EtOH	Antimicrobial	[50,63]
14.	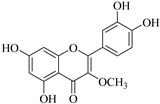 3-*O*-methylquercetin	Flavonol	*E. lunulata*	Aerial, EtOH	Antiproliferative	[25,26]
15.	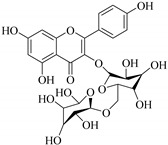 kaempferol 3-*O*-rutinoside	Glucosidic flavonol	*E. larica*, *E. virgata*, *E. mgalanta*, *E. helioscopia*, *E. bivonae*, *E. ebracteolata*	Whole plant, EtOH	Not evaluated	[56,64,65,66]
16.	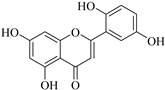 5,7,2′,5′-tetrahydroxyflavone	Flavone	*E. lunulata*	Aerial, EtOH	Antiproliferative	[25,26]
17.	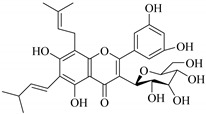 hirtaflavonoside-B (5,7,3′,4′ –trihyroxy-6-(3,3 –dimethyl allyl)-8-9-iso-butenyl)-flavonol-3-*O*-*β*-d-glucosidase)	Glucosidic flavonol	*E. hirta*	Roots, MeOH	Inhibitory activity (α-glucosidase)	[49]
18.	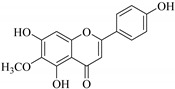 6-methoxyapigenin	Flavone	*E. larica*, *E. lunulata*	Aerial, EtOH	Antiproliferative	[29,67]
19.	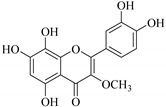 5,7,8,3′,4′-pentahydroxy-3-methoxyflavone	Flavone	*E.* *retusa*	Whole plant, MeoH	Not evaluated	[48]
20.	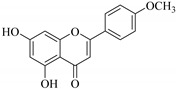 acacetin	Flavone	*E. bivonae*	Roots, MeOH	Not evaluated	[66]
21.	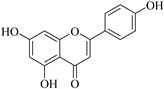 apigenin	Flavone	*E. lunulata*, *E. condylocarpa*	Aerial, EtOH	Antiproliferative, antioxidant, anti-tumour, anti-inflammatory, antibacterial, antiproliferative	[25,26,68]
22.	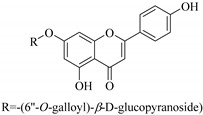 apigenin-7-*O*-(6′′- *O*-galloyl)-*β*-d-glucopyranoside	Glucosidic flavone	*E. humifusa*	Whole plant, EtOH	Anti-HBV	[58]
23.	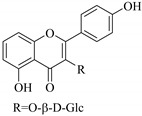 apigenin-7-*O*-*β*-d-glucoside	Glucosidic flavone	*E. lunulata*, *E. humifusa*	Aerial, EtOH	Anti-HBV	[26,54,58]
24.	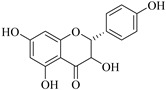 aromadendrin	Flavonol	*E. cuneate*	Aerial, EtOH	Antiulcerogenic	[69]
25.	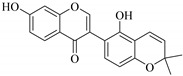 glabrone	Flavone	*E. helioscopia*	Whole plant, EtOH	Not evaluated	[56]
26.	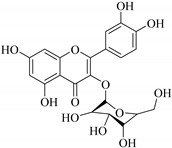 hyperoside	Flavone	*E. lunulata*	Aerial, EtOH	Antiproliferative	[26,70]
27.	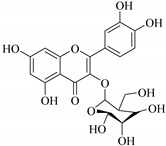 hyperin	Glucosidic flavonol	*E. lunulata*	Whole plant, C_3_H_6_O	Antiproliferative	[60]
28.	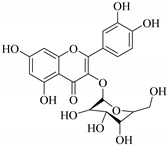 isoquercetin	Glucosidic flavonol	*E. lunulata*, *E. tirucalli*, *E. ebracteolata*	Aerial, EtOH	Antiproliferative	[25,26,50,71]
29.	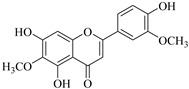 jaceosidin	Flavone	*E. lunulata*	Aerial, EtOH	Antiproliferative	[26,67]
30.	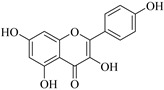 kaempferol	Flavonol	*E. guyoniana*, *E. allepica*, *E*. *charnaesyce*, *E. rnagalanta*, *E. virgate*, *E. lunulata*, *E. hirta*, *E. wallichii*	Aerial; C_3_H_6_O:MeOH, whole plant; Me_2_CO_2_, Leaf; EtOH	Not evaluated	[26,29,55,72,73,74]
31.	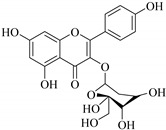 kaempferol 3-*O*-glucoside	Glucosidic flavonol	*E. guyoniana*, *E. rnagalanta*, *E*. *charnaesyce*, *E. virgata*	Aerial, C_3_H_6_O:MeOH, Leaf; EtOH	Not evaluated	[29,72]
32.	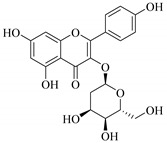 kaempferol 3-*O*-*β*-d-glucopyranoside	Glucosidic flavonol	*E. altotibetic*, *E. retusa*	Whole plant		[54]
33.	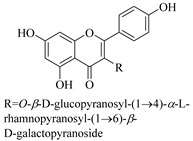 kaempferol 3-*O*-*β*-d-glucopyranosyl-(1→4)-*α*-l-rhamnopyranosyl-(1→6)-*β*-d-galactopyranoside	Carbohydrate flavonol	*E. ebracteolata*	Aerial, EtOH	Not evaluated	[59]
34.	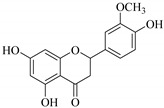 isorhamnetin	Flavanone	*E. hirta*, *E. guyoniana*, *E. charnaesyce*, *E. rnagalanta*, *E. amygdaloides*	Aerial, C_3_H_6_O:MeOH, Leaf; EtOH	Not evaluated	[29,72,75]
35.	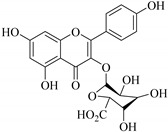 kaempferol-3-*O*-*β*-d-glucuronide	Glucosidic flavonol	*E. lathyris*	Aerial, EtOH	Cytotoxic	[57]
36.	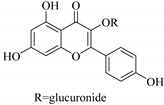 kaempferol-3-glucuronide	Glucosidic flavonol	*E. lathyris*	Aerial, MeoH	Not evaluated	[51,57]
37.	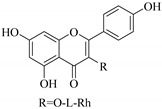 kaempferol-3-l-rhamnoside	Carbohydrate flavonol	*E. lunulata*	Aerial, EtOH	Antiproliferative	[26,70]
38.	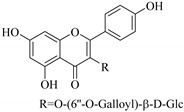 kaempferol-3-*O*-(6′′-galloyl)-*β*-d-glucoside	Carbohydrate flavonol	*E. lunulata*, *E. fischeriana*, *E. esula*	Aerial, EtOH	Antiproliferative	[26,76]
39.	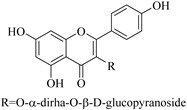 kaempferol-3-*O*-*α*-rhamnoside-*O*-*β*-d-glucopyranoside	Carbohydrate flavonol	*E. bivonae*	Roots, MeOH	Not evaluated	[66]
40.	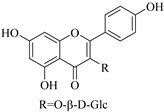 kaempferol-3-*O*-*β*-d-glucoside	Glucosidic flavonol	*E. lunulata*, *E. fischeriana*, *E. esula*	Aerial, EtOH	Antiproliferative	[26,76]
41.	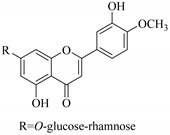 4′-*O*-methoxy-luteolin-7-*O*-rhamnoglucoside	Carbohydrate flavonol	*E. cuneate*	Aerial, EtOH	Antiulcerogenic	[69]
42.	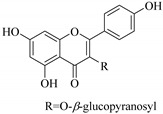 kaempferol-3-*β*-d glucopyranosyl	Glucosidic flavonol	*E.* *retusa*	Whole plant, MeoH	Not evaluated	[48]
43.	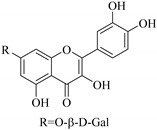 kaempferol-7-*O*-*β*-d-glucoside	Glucosidic flavonol	*E. lunulata*, *E. fischeriana*, *E. esula*	Aerial, EtOH	Antiproliferative	[26,76]
44.	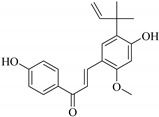 licochalcone A	Chalcone	*E. helioscopia*	Whole plant, EtOH	Not evaluated	[56]
45.	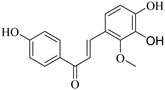 licochalcone B	Chalcone	*E. helioscopia*	Whole plant, EtOH	Not evaluated	[56]
46.	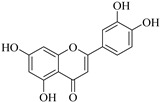 luteolin	Flavone	*E. lunulata*, *E. hirta*, *E. humifusa*, *E. bivonae*	Aerial, whole plant, roots, EtOH,	Antiproliferative, Anti-HBV	[25,26,58,66,73]
47.	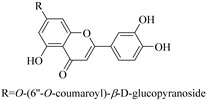 luteolin-7-*O*-(6′′-*O*-coumaroyl)-*β*-d-glucopyranoside	Glucosidic flavonol	*E. humifusa*	Whole plant, EtOH	Anti-HBV	[58]
48.	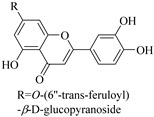 luteolin-7-*O*-(6′′-*O*-trans- feruloyl)-*β*-d-glucopyranoside	Glucosidic flavonol	*E. humifusa*	Whole plant, EtOH	Anti-HBV	[58]
49.	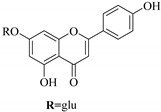 luteolin-7-*O*-*β*-d-glucopyranoside	Glucosidic flavonol	*E. humifusa*	Whole plant, EtOH	Anti-HBV	[58]
50.	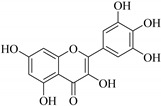 myricetin	Flavonol	*E. lunulata*, *E. wallichii*	Aerial, EtOH	Antiproliferative	[25,26,74]
51.	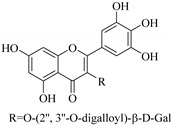 myricetin-3-*O*-(2′′,3′′-digalloyl)-*β*-d-galactopyranoside	Carbohydrate flavone	*E. lunulata*	Aerial, EtOH	Antiproliferative	[25,26]
52.	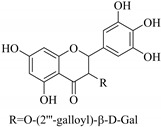 myricetin-3-*O*-(2′′-galloyl)-*β*-d-galactopyranoside	Carbohydrate flavone	*E. lunulata*	Aerial, EtOH	Antiproliferative	[25,26]
53.	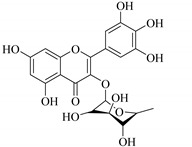 myricitrin	Glucosidic flavone	*E. lunulata*	Aerial, EtOH	Antiproliferative	[25,26]
54.	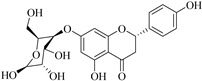 naringenin-7-*O*-*β*-d-glucoside	Glucosidic flavone	*E. lunulata*	Aerial, EtOH		[26,54]
55.	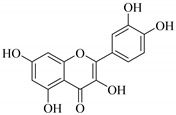 quercetin	Flavonol	*E. guyoniana*, *E. stenoclada*, *E. hirta*, *E. neriifolia*, *E*. *charnaesyce*, *E. rnagalanta*, *E. virgate*, *E. lunulata*, *E. humifusa*, *E. helioscopia*, *E. tirucalli*	Aerial, leaves, C_3_H_6_O:MeOH, Leaf; EtOH	Antiproliferative, anti-HBV, antidiarrheal, anticancer, antimalarial, antibacterial, antifungal	[26,29,50,53,58,62,67,72,77]
56.	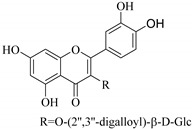 quercetin 3-*O*-(2′,3′-digalloyl)-*β*-d-galactopyranoside	Carbohydrate flavonol	*E. lunulata*	Whole plant, C_3_H_6_O	Antiproliferative	[60]
57.	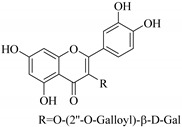 quercetin 3-*O*-(2′-galloyl)-*β*-d-galactopyranoside	Carbohydrate flavonol	*E. lunulata*	Whole plant, C_3_H_6_O	Antiproliferative	[60]
58.	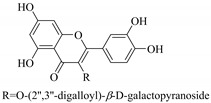 quercetin 3-*O*-(2″,3″-digalloyl)-*β*-d-galactopyranoside	Carbohydrate flavonol	*E. lunulata*	Roots, MeOH	Antiproliferation activity	[78]
59.	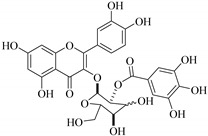 quercetin 3-*O*-(2″-galloyl)-*β*-d-galactopyranoside	Carbhydrate flavonol	*E. lunulata*	Roots, MeOH	Antiproliferation activity	[78]
60.	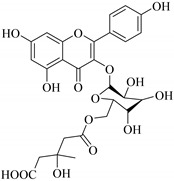 quercetin 3-*O*-6′′-(3-hydroxyl-3-methylglutaryl)-*β*-d-glucopyranoside	Glucosidic flavonol	*E. ebracteolata*	Aerial, EtOH	Not evaluated	[59]
61.	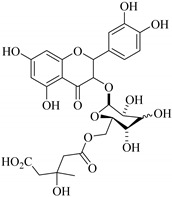 quercetin 3-*O*-6′-(3hydroxyl-3-methylglutaryl)-*β*-d-glucopyranoside	Glucosidic flavonol	*E. ebracteolata*	Aerial, MeOH	Not evaluated	[78,79]
62.	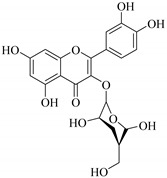 quercetin 3-*O*-glucoside	Glucosidic flavonol	*E. guyoniana*, *E. charnaesyce*, *E. virgate*, *E. paralias*, *E. condylocarpa*	Aerial, whole plant, C_3_H_6_O:MeOH, Leaf; EtOH	Anticancer against colon, breast, hepato cellular and lung cancer cell lines, inhibitory activities	[29,52,68,72]
63.	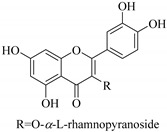 quercetin 3-*O*-*α*-l-rhamnopyranoside	Carbohydrate flavonol	*E. heterophylla*	Aerial, EtOH	Not evaluated	[46]
64.	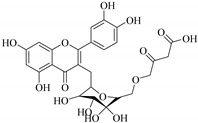 quercetin 3-*O*-*β*-d-6′′-malonate	Carbohydrate flavonol	*E. heterophylla*	Aerial, EtOH	Not evaluated	[46]
65.	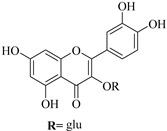 quercetin 3-*O*-*β*-d-glucopyranoside	Glucosidic flavonol	*E. paralias*, *E. humifusa*, *E. microsciadia*, *E. heterophylla*, *E. ebracteolata*	Whole plant, Aerial, EtOH	Ant-HBV	[45,52,58,71]
66.	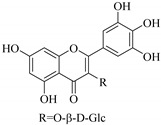 quercetin-3-*O*-*β*-glucuronic	Glucosidic flavonol	*E. lunulata*, *E. esula*	Aerial, EtOH	Antiproliferative	[26,80]
67.	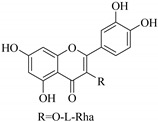 quercetin-3-l-rhamnoside	Carbohydrate flavonol	*E. lunulata*	Aerial, EtOH	Antiproliferative	[26,70]
68.	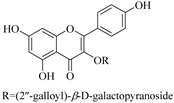 quercetin-3-*O*- (2′′-galloyl)-*β*-d-galactopyranoside	Glucosidic flavonol	*E. lunulata*	Aerial, EtOH	Antiproliferative	[26,70]
69.	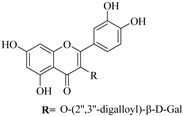 quercetin-3-*O*-(2′′,3′′- digalloyl)-*β*-d-galactopyranoside	Glucosidic flavonol	*E. lunulata*	Aerial, EtOH	Antiproliferative	[26,70]
70.	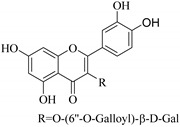 quercetin-3-*O*-(6′′-galloyl)-*β*-d-galactopyranoside	Carbohydrate flavonol	*E. lunulata*	Aerial, EtOH		[26,54]
71.	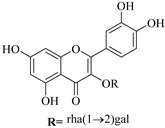 quercetin-3-*O*-*α*-l-rhamnosyl (1→6)-*β*-d-galactoside	Carbohydrate flavonol	*E. humifusa*	Whole plant, EtOH	Anti-HBV	[58]
72.	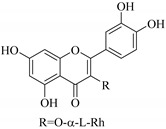 quercetin-3-*O*-*α*-rhamnoside	Carbohydrate flavonol	*E. hirta*	Whole plant, MeOH	Anti-snake venom activity	[81]
73.	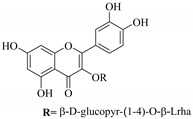 quercetin-3-*O*-*β*- d-glucopyranosyl- (1-4)-*O*-*α*-l-rhamnopyranoside	Carboydrate flavonol	*E.drancunculoides*	Aerial, EtOH	Cytotoxic	[82]
74.	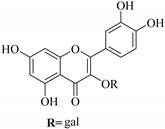 quercetin-3-*O*-*β*-d-galactoside	Glucosidic flavonol	*E. humifusa*,	Whole plant, EtOH,	Anti-HBV, cytotoxic	[58]
75.	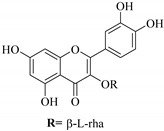 quercetin-3-*O*-*β*-l-rhamnoside	Carbohydrate flavonol	*E. lunulata*, *E. fischeriana*, *E. esula*	Aerial, EtOH	Antiproliferative	[26,76]
76.	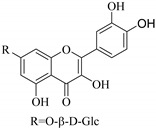 quercetin-7-*O*-*β*-d-glucoside	Glucosidic flavonol	*E. lunulata*, *E. fischeriana*, *E. esula*	Aerial, EtOH	Antiproliferative	[26,76]
77.	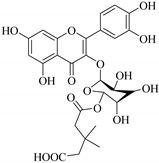 quercetin 3-*O*-6′′-(3-hydroxy-3-methylglutaryl)-*β*-d-glucopyranoside	Glucosidic flavonol	*E. ebracteolata*	leaves		[83]
78.	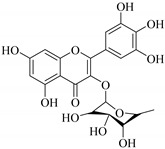 quercitrin	Glucosidic flavonol	*E. stenoclada*, *E. tirucalli*	Aerial, MeOH, EtOH	Antiproliferative, antibacterial, antifungal	[50,53]
79.	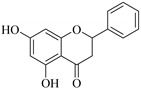 pinocembrin	Flavone	*E. hirta*	Aerial, EtOH	Not evaluated	[75]
80.	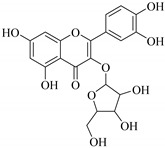 rhamnetin-3-*α*-arabinofuranoside	Carbohydrate flavonol	*E. lathyris*, *E-amygdaloides*	Aerial, EtOH	Not evaluated	[51,75]
81.	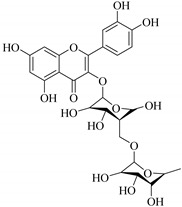 rutin	Glucosidic flavonol	*E. guyoniana*, *E. charnaesyce*, *E. rnagalanta*, *E. virgate*, *E. tirucalli*, *E. ebracteolata*	Aerial, C_3_H_6_O:MeOH, Leaf; EtOH	Antibacterial, antifungal	[29,50,71,72]
82.	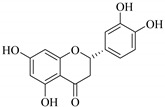 eriodictyol	Flavone	*E. matabelensis*	Stems, MeOH	Antiproliferative	[84]
83.	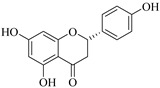 naringenin	Flavone	*E. matabelensis*	Stems, MeOH	Antiproliferative	[84]
84.	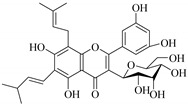 5,7,3′,4′ -trihyroxy-6-(3,3 –dimethyl allyl)-8-9-iso-butenyl)-flavonol-3-C-*β*-d-glucosidase	Glucosidic flavonol	*E. hirta*	Aerial, MeOH	Inhibitory activity (α-glucosidase)	[85]
85.	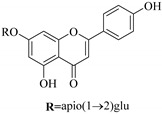 apigenin-7-*O*-*β*-d-apiofuranosyl(1→2)-*β*-d-glucopyranoside	Glucosidic flavonol	*E. humifusa*	Whole plant, EtOH	Anti-HBV	[58]
86.	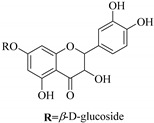 isoaromadendrin-7-*O*-*β*-d-glucopyranoside (isosinemsin)	Glucosidic flavonol	*E. cuneata*	Whole plant, EtOH	Hypertensive	[86]

## Data Availability

Not applicable.

## References

[1] De Montellano B.O. (1975). Empirical Aztec medicine. Science.

[2] Madeleine E., Olwen M., Grace C., Haris S.L., Niclas N., Henrik T., Nina R. (2015). Global medicinal uses of *Euphorbia* L. (*Euphorbiaceae*). J. Ethnopharmacol..

[3] Hooper M. (2002). Major Herbs of Ayurveda.

[4] Kemboi D., Xolani P., Moses L., Jacqueline T. (2020). A review of the ethnomedicinal uses, biological activities and triterpenoids of *Euphorbia* species. Molecules.

[5] Islam M.T., Mata O., Aguiar R.S., Paz M.C.J., Alencar M.B., Melo-Cavalcante A.C. (2016). Therapeutic potential of essential oils focusing on diterpenes. Phytother. Res..

[6] Mihail A., Viktorija M., Liljana K.G., Liljana G.T. (2019). Review of the anticancer and cytotoxic activity of some species from the genus *Euphorbia*. Agric. Conspec. Sci..

[7] Muhammad A., Abdul S., Muhammad J., Farhat U., Muhammad O., Ikram U., Jawad A., Muhammad S. (2019). Flavonoids as prospective neuroprotectants and their therapeutic propensity in aging associated neurological disorders. Front. Aging Neurosci..

[8] Scholz A., Engler A. (1964). Euphorbiaceae. Syllabus der Planzenfamilien.

[9] Webster G.L. (1994). Classification of the *Euphorbiaceae*. Ann. Mol. Bot. Gard..

[10] Andrea V., Judit H. (2014). *Euphorbia* diterpenes: Isolation, structure, biological activity, and synthesis (2008–2012). Chem Rev..

[11] Nabavi S.M., Samec D., Tomczyk M., Milella L., Russo D., Habtemariam S., Suntar I., Rastrelli L., Daglia M., Xiao J. (2018). Flavonoid biosynthetic pathways in plants: Versatile targets for metabolic engineering. Biotechnol. Adv..

[12] Scarano A., Chieppa M., Santino A. (2018). Looking at flavonoid biodiversity in horticultural crops: A colored mine with nutritional benefits. Plants.

[13] Sudhakaran M., Sardesai S., Dose A.I. (2019). Flavonoids: New frontier for immuno-regulation and breast cancer control. Antioxidants.

[14] Pretorius J.C. (2003). Flavonoids: A review of its commercial application potential as anti-infective agents. Anti-Infect. Agents Curr. Med. Chem..

[15] Zhao K., Yuan Y., Lin B., Miao Z., Li Z., Guo Q., Lu N. (2018). LW-215, a newly synthesized flavonoid, exhibits potent anti-angiogenic activity in vitro and in vivo. J. Pharmacol. Sci..

[16] Liu J., Wang X., Yong H., Kan J., Jin C. (2018). Recent advances in flavonoid-grafted polysaccharides: Synthesis, structural characterization, bioactivities and potential applications. Int. J. Biol. Macromol..

[17] Camero C.M., Germanò M.P., Rapisarda A., D’Angelo V., Amira S., Benchikh F., Braca A., De Leo M. (2018). Anti-angiogenic activity of iridoids from *Galium tunetanum*. Rev. Bras. Farm..

[18] Patel K., Kumar V., Rahman M., Verma A., Patel D.K. (2018). New insights into the medicinal importance, physiological functions and bioanalytical aspects of an important bioactive compound of foods ‘Hyperin’: Health benefits of the past, the present, the future. Beni-Suef Univ. J. Basic Appl. Sci..

[19] Zamora-Ros R., Guinó E., Alonso M.H., Vidal C., Barenys M., Soriano A., Moreno V. (2015). Dietary flavonoids, lignans and colorectal cancer prognosis. Sci. Rep..

[20] Avtar C., Bhawna G. (2019). Chemistry and Pharmacology of flavonoids—A review. Indian J. Pharm. Edu. Res..

[21] Reale G., Russo G.I., di Mauro M., Regis F., Campisi D., Giudice A.L., Marranzano M., Ragusa R., Castelli T., Cimino S. (2018). Association between dietary flavonoids intake and prostate cancer risk: A case-control study in Sicily. Complement. Ther. Med..

[22] Rienks J., Barbaresko J., Nothlings U. (2017). Association of isoflavone biomarkers with risk of chronic disease and mortality: A systematic review and meta-analysis of observational studies. Nutr. Rev..

[23] Darband S.G., Kaviani M., Yousefi B., Sadighparvar S., Pakdel F.G., Attari J.A., Mohebbi I., Naderi S., Majidinia M. (2018). Quercetin: A functional dietary flavonoid with potential chemo-preventive properties in colorectal cancer. J. Cell. Physiol..

[24] Danihelová M., Viskupiˇcová J., Šturdík E. (2012). Lipophilization of flavonoids for their food, therapeutic and cosmetic applications. Acta Chim..

[25] Yang Z.-G., Jia L.-N., Shen Y., Ohmura A., Kitanaka S. (2011). Inhibitory effects of constituents from *Euphorbia lunulata* on differentiation of 3T3-L1 cells and nitric oxide production in RAW264.7 cells. Molecules.

[26] Yuwei W., Xiao Y., Lingna W., Fang Z., Yongqing Z. (2020). Research progress on chemical constituents and anticancer pharmacological activities of *Euphorbia lunulata* Bunge. Bio. Med. Res. Int..

[27] Caballero B., Trugo L., Finglas P. (2003). Encyclopedia of Food Sciences and Nutrition.

[28] Sotnikova O.M., Chagovets R.K., Litvinenko V.I. (1968). New flavanone compounds from *Euphorbia stepposa*. Chem. Nat. Compd..

[29] Ulubelen A., Öksüz S., Halfon B., Aynehchi Y., Mabry T.J. (1983). Flavonoids from *Euphorbia larica*, *E. virgata*, *E. chamaesyce* and *E. magalanta*. J. Nat. Prod..

[30] Dieter T. (2006). Significance of flavonoids in plant resistance: A review. Environ. Chem. Lett..

[31] Ali S., Behrad D., Farzaneh H., Azadeh M., Antoni S., Seyed F., Leo R., Seyed M., Anupam B. (2017). Therapeutic potential of flavonoids in inflammatory bowel disease: A comprehensive review. World J. Gastroenterol..

[32] Mariam A., Samson M., Elizabeth V., Sharon V., Peter K., Alena L., Dietrich B. (2019). Flavonoids in cancer and apoptosis. Cancers.

[33] Bathelemy N., Ghislain W., Justin K., Pantaleon A., Tchoukoua A., Aimé G., Igor K., Bonaventure T., Berhanu M., Victor K. (2019). Flavonoids and related compounds from the medicinal plants of Africa. J. Med. Plant. Res..

[34] Panche A.N., Diwan A.D., Chandra S.R. (2013). Flavonoids: An overview. Sci. World J..

[35] Shashank K., Abhay K. (2013). Chemistry and biological activities of flavonoids: An Overview. Sci. World J..

[36] Nigel C., Renee J. (2011). Flavonoids and their glycosides, including anthocyanins. Nat. Prod. Rep..

[37] Goel G., Makkar S., Francis G., Becker K. (2007). Phorbol esters: Structure, biological activity, and toxicity in animals. Int. J. Toxicol..

[38] Shi Q.W., Su X.H., Kiyota H. (2008). Chemical and pharmacological research of the plants in genus *Euphorbia*. Chem. Rev..

[39] Rojas R., Tafolla-Arellano J.C., Martínez-Ávila G.C.G. (2021). *Euphorbia antisyphilitica* Zucc: A Source of Phytochemicals with Potential Applications in Industry. Plants.

[40] Yang T., Jun H., Yu Y., Wen-Wen L., Cong-Yuan X., Jie-Kun X., Wei-Ku Z. (2021). *Euphorbia ebracteolata* Hayata (Euphorbiaceae): A systematic review of its traditional uses, botany, phytochemistry, pharmacology, toxicology, and quality control. Phytochemistry.

[41] Agati G., Azzarello E., Pollastri S., Tattini M. (2012). Flavonoids as antioxidants in plants: Location and functional significance. Plant Sci..

[42] Middleton E. (1984). The flavonoids. Trends Pharmacol. Sci..

[43] Murphy K., Chronopoulos A., Singh I. (2003). Dietary flavanols and procyanidin oligomers from cocoa (Theobroma cacao) inhibit platelet function. Am. J. Clin. Nutr..

[44] Ke L., Hang F., Peipei Y., Lingguang Y., Qiang X., Xiang L., Liwei S., Yujun L. (2018). Structure-activity relationship of eight high content flavonoids analyzed with a preliminary assign-score method and their contribution to antioxidant ability of flavonoids-rich extract from *Scutellaria*. Arab. J. Chem..

[45] Syed M., Ghanadiana A., Suleiman A., Sumaira H., Omer A., Jean J. (2012). Flavonol glycosides from *Euphorbia microsciadia* Bioss with their immunomodulatory activities. Iran. J. Pharm. Res..

[46] De-freitsa T.J.B., Silva A.J., Kuster R. (2019). Isolation and characterization of polyphenols from *Euphorbia heterophylla* L. (Euphorbiaceae) leaves. Rev. Fitos.

[47] Zhang W.J. (2016). Study on Chemical Constituents and Antitumor Activity of Euphorbia Lunulata Bge.

[48] Laila A.-G. (2011). Study on Flavonoids and Triterpenoids content of some *Euphorbiaceae* plants. J. Life Sci..

[49] Sheliya M.A., Rayhana B., Ali A., Pillai K.K., Aeri V., Sharmam R. (2015). Inhibition of α-glucosidase by new prenylated flavonoids from *Euphorbia hirta* l. herbs. J. Ethnopharmacol..

[50] Maria D., Luziene A.C., Emily C., Bruno O., M´arcia V., Lívia N.C., Renata M. (2021). Bioactivity flavonoids from roots of *Euphorbia tirucalli* L.. Phytochem. Lett..

[51] Seham S., Raba M., Ahmed F., Nadia M., Sameh F., Mohamed N., Usama R.A., Elham A. (2021). Cytotoxic activity and metabolic profiling of fifteen *Euphorbia* Species. Metabolites.

[52] Safwat N.A., Kashef M.T., Aziz R.K., Amer K.F., Ramadan M.A. (2017). Quercetin 3-O-glucoside recovered from the wild Egyptian Sahara plant, *Euphorbia paralias* L., inhibits glutamine synthetase and has antimycobacterial activity. Tuberculosis.

[53] Chaabi M., Freund-Michel V., Frossard N., Randriantsoa A., Andriantsitohaina R., Lobstein A. (2007). Anti-proliferative effect of *Euphorbia stenoclada* in human airway smooth muscle cells in culture. J. Ethnopharmacol..

[54] Li R., Wang J., Wu H.X., Li L., Wang N.L. (2011). Isolation, identification and activity determination of antioxidant active components in *Euphorbia lunulata* Bunge. J. Shenyang Pharm. Univ..

[55] Sevil O., Faliha G., Long-Ze L., Roberto R., Gil J.M., Pezzuto M., Geoffrey A.C. (1996). Aleppicatines A and B from *Euphorbia allepica*. Phytochemistry.

[56] Wen Z., Yue-Wei G. (2006). Chemical studies on the constituents of the chinese medicinal herb *Euphorbia helioscopia* L.. Chem. Pharm. Bull..

[57] Dumkow K. (1969). Kaempferol-3-glucuronide and quercetin-3-glucuronide, principal flavonoids of *Euphorbia lathyris* L. and their separation on acetylated polyamide. Z. Naturforsch.

[58] Ying T., Li-Min S., Xi-Qiao L., Bin Li Q., Jun-Xing D. (2010). Anti-HBV active flavone glucosides from *Euphorbia humifusa* Willd. Fitoterapia.

[59] Liu X., Ye W., Yu B., Zhao S., Wu H., Che C. (2004). Two new flavonol glycosides from *Gymnema sylvestre* and *Euphorbia ebracteolata*. Carbohydr. Res..

[60] Tadahiro N., Li-Yan W., Kouji K., Susumu K. (2005). Flavonoids that mimic human ligands from the whole plants of *Euphorbia lunulata*. Chem. Pharm. Bull..

[61] Veena S., Pracheta J. (2017). Extraction, isolation and identification of flavonoid from *Euphorbia neriifolia* leaves. Arab. J. Chem..

[62] Cheng J., Han W., Wang Z., Shao Y., Wang Y., Zhang Y., Li Z., Xu X., Zhang Y. (2015). Hepatocellular carcinoma growth is inhibited by *Euphorbia helioscopia* L. extract in nude mice xenografts. Bio. Med. Res. Int..

[63] Abu-reidah I.M., Ali-shtayeh M.S., Jamous R.M., Arr´aez-rom´an D., Segura-carretero A. (2015). HPLC–DAD–ESI-MS/MS screening of bioactive components from *Rhus coriaria* L. (Sumac) fruits. Food Chem..

[64] Markham K.R., Ternai B. (1976). 13C NMR of flavonoids-ii flavonoids other than flavone and flavonol aglycones. Tetrahedron.

[65] Ahn B.T., Lee K.S. (1996). Phenolic compounds from *Euphorbia ebracteolata*. Saengyak Hakhoechi.

[66] Heba I., Abd E.-M. (2015). Flavonoid and phenolic acid compounds in *Euphorbia bivonae* Steud roots. Curr. Sci. Int..

[67] Liu C., Sun H., Wang W.T. (2015). Study on chemical constituents of *Euphorbia lunulata* bge. J. Chin. Med. Mater..

[68] Hassana G.K., Omera M.A., Babadoust S., Najata D.D. (2014). Flavonoids from *Euphorbia condylocarpa* roots. Int. J. Chem. Bio. Sci..

[69] Amani S.A., Nabilah A., John E., Reham M., Mohamed E. (2013). Antiulcerogenic activities of the extracts and isolated flavonoids of *Euphorbia cuneata* Vahl. Phytother. Res..

[70] Shang T.M., Wang L., Liang X.T. (1979). Study on the chemical constituents of *Euphorbia Lunulata*. J. Chem..

[71] Lee S.C., Ahn B.T., Park W.Y., Lee S.H., Ro J.S., Lee K.S., Ryu E.K. (1992). Pharmacognostic study on *Euphorbia ebracteolata*. Flavonoid constituents. Korean J. Pharmacogn..

[72] Tarek B., Hichema A., Khalfallaha A., Kabouchea Z., Kabouchea I., Jaime B., Christian B. (2010). A New alkaloid and flavonoids from the aerial parts of *Euphorbia guyoniana*. Nat. Prod. Commun..

[73] Wu Y., Qu W., Geng D., Liang J.-Y., Luo Y.-L. (2012). Phenols and flavonoids from the aerial part of *Euphorbia hirta*. Chin. J. Nat. Med..

[74] Abida T., Ismat N., Hifsa M. (2009). Isolation of flavonols from Euphorbia wallichii by preparative High Performance Liquid Chromatography. Nat. Sci..

[75] Mueller R., Pohl R. (1970). Flavonol glycosides of *Euphorbia amygdaloides* and their quantitative determination at various stages of plant development. 5. Flavonoids of native *Euphorbiaceae*. Planta Med..

[76] Chai G.S. (2013). Study on the chemical constituents of the seeds of *Euphorbia Fischeriana* Steud and *Euphorbia Esula Linn.* Qiqihar University, Qiqihar, China. Chemo-prevention. Food Chem..

[77] Salehi B., Iriti M., Vitalini E., Antolak H., Pawlikowska E., Kręgiel D., Sharifi-rad J., Oyeleye S.I., Ademiluyi A.O., Czopek K. (2019). *Euphorbia*-derived natural products with potential for use in health maintenance. Biomolecules.

[78] Ozbílgín S., Saltan G. (2012). Uses of some *Euphorbia* species in traditional medicine in turkey and their biological activities. Turk. J. Pharm. Sci..

[79] Liu Z.G., Li Z.L., Bai J., Meng D.L., Li N., Pei Y.H., Zhao F., Hua H.M. (2014). Anti-inflammatory diterpenoids from the roots of *Euphorbia ebracteolata*. J. Nat. Prod..

[80] Halaweish F., Kronberg S., Rice J. (2003). Rodent and ruminant ingestive response to flavonoids in *Euphorbia esula*. J. Chem. Ecol..

[81] Kadiyala G., Anbarasu K., Kadali R., Jayanthi S., Vishwanath B.S., Gurunathan J. (2016). Quercetin-3-O-rhamnoside from *Euphorbia hirta* protects against snake venom induced toxicity. Biochim. Biophys. Acta.

[82] Noori M., Chehreghani A., Kaveh M. (2009). Flavonoids of 17 species of *Euphorbia* (Euphorbiaceae). Iran. Toxicol. Environ. Chem..

[83] Byung T.A., Kapjin O., Jai S.R., Kyong S.L. (1996). A New Flavonoid from *Euphorbia ebracteolata*. Planta Med..

[84] Reham H., Norbert K., Peter W., Ágnes K., István Z., Péter O., László T., Judit H., Andrea V. (2019). Isolation and pharmacological investigation of compounds from *Euphorbia matabelensis*. Nat. Prod. Commun..

[85] Martinez V.M., Ramirez A.T., Lazcano M., Bye R. (1999). Anti-inflammatory Active Compounds from then-hexane extract of *Euphorbia hirta*. J. Mex. Chem. Soc..

[86] Bahar A., Tawfeq A., Jaber S.m., Kehel T. (2005). Isolation, antihypertensive activity and structure activity relationship of flavonoids from medicinal plants. Indian J. Chem..

[87] Ko W.C., Wang H.L., Lei C.B., Shih C.H., Chung M.I., Lin C.N. (2002). Mechanisms of relaxant action of 3-*O*-methylquercetin in isolated guinea pig trachea. Planta Medica.

[88] Haggag E.G., Abou-Moustafa M.A., Boucher W., Theoharides T.C. (2003). The effect of herbal water-extract on histamine release from mast cells and on allergic asthma. J. Herb. Pharmacother..

[89] Jiang S. (2011). The Inhibition of Lung Cancer Active Ingredient Extracts of Euphorbia Lunulata and Its Mechanism.

[90] Gao F., Fu Z., Tian H., He Z. (2013). The *Euphorbia lunulata* extract inhibits proliferation of human hepatoma Hep-G2 cells and induces apoptosis. J. BUON.

[91] Hameed A., Hafizur R.M., Hussain N. (2018). Eriodictyol stimulates insulin secretion through cAMP/PKA signaling pathway in mice islets. Eur. J. Pharmacol..

[92] Campos P.M., Prudente A.S., Horinouchi C.D. (2015). Inhibitory effect of GB-2a (I3-naringenin-II8-eriodictyol) on melanogenesis. J. Ethnopharmacol..

[93] Lee J.K. (2011). Anti-inflammatory effects of eriodictyol in lipopolysaccharide-stimulated raw 264.7 murine macrophages. Arch. Pharm. Res..

[94] Whelan L.C., Ryan M.F. (2003). Ethanolic extracts of Euphorbia and other ethnobotanical species as inhibitors of human tumour cellgrowth. Phytomedicine.

[95] Wongprayoon P., Charoensuksai P. (2018). Cytotoxic and anti-migratory activities from hydroalcoholic extract of *Euphorbia lactea* Haw against HN22 cell line. Thai J. Pharm. Sci..

[96] Villanueva J., Quirós L.M., Castañón S. (2015). Purification and partial characterization of a ribosome-inactivating protein from the latex of *Euphorbia trigona* Miller with cytotoxic activity toward human cancer cell lines. Phytomedicine.

[97] Waczuk E.P., Kamdem J.P., Abolaji A.O., Meinerz D.F., Bueno D.C., do Nascimento Gonzaga T.K., do Canto Dorow T.S., Boligon A.A., Athayde M.L., da Rocha J.B.T. (2015). *Euphorbia tirucalli* aqueous extract induces cytotoxicity, genotoxicity and changes in antioxidant gene expression in human leukocytes. Toxicol. Res..

[98] Hou D.X., Kumamoto T. (2010). Flavonoids as protein kinase inhibitors for cancer chemoprevention: Direct binding and molecular modeling. Antioxid. Redox Signal..

[99] Lolli G., Cozza G., Mazzorana M., Tibaldi E., Cesaro L., Donella-Deana A., Pinna L.A. (2012). Inhibition of protein kinase CK2 by flavonoids and tyrphostins. A structural insight. Biochemistry.

[100] Yokoyama T., Kosaka Y., Mizuguchi M. (2015). Structural insight into the interactions between death-associated protein kinase 1 and natural flavonoids. J. Med. Chem..

[101] Peng H.L., Huang W.C., Cheng S.C., Liou C.J. (2018). Fisetin inhibits the generation of inflammatory mediators in interleukin-1β-induced human lung epithelial cells by suppressing the Nf-κb and Erk1/2 pathways. Int. Immunopharmacol..

[102] Atashpour S., Fouladdel S., Movahhed T.K., Barzegar E., Ghahremani M.H., Ostad S.N., Azizi E. (2015). Quercetin induces cell cycle arrest and apoptosis in CD133+ cancer stem cells of human colorectal HT29 cancer cell line and enhances anticancer effects of doxorubicin. Iran. J. Basic Med. Sci..

[103] Yang P.-W., Lu Z.-Y., Pan Q., Chen T.-T., Feng X.-J., Wang S.-M., Pan Y.-C., Zhu M.-H., Zhang S.-H. (2019). MicroRNA-6809–5p mediates luteolin-induced anticancer effects against hepatoma by targeting flotillin. Phytomedicine.

[104] Guo Y.Q., Tang G.H., Lou L.L., Li W., Zhang B., Liu B., Yin S. (2018). Prenylated flavonoids as potent phosphodiesterase-4 inhibitors from Morus alba: Isolation, modification, and structure-activity relationship study. Eur. J. Med. Chem..

[105] Choene M., Motadi L. (2016). Validation of the antiproliferative effects of *Euphorbia tirucalli* extracts in breast cancer cell lines. Mol. Biol..

[106] Devi K.P., Rajavel T., Nabavi S.F., Setzer W.N., Ahmadi A., Mansouri K., Nabavi S.M. (2015). Hesperidin: A promising anticancer agent from nature. Ind. Crops Prod..

[107] Ersoz M., Erdemir A., Duranoglu D., Uzunoglu D., Arasoglu T., Derman S., Mansuroglu B. (2019). Comparative evaluation of hesperetin loaded nanoparticles for anticancer activity against C6 glioma cancer cells. Artif. Cells Nanomed. Biotechnol..

[108] Alsayari A., Muhsinah A.B., Hassan M.Z., Ahsan M.J., Alshehri J.A., Begum N. (2019). Aurone: A biologically attractive scaffold as anticancer agent. Eur. J. Med. Chem..

[109] Yahfoufi N., Alsadi N., Jambi M., Matar C. (2018). The immunomodulatory and anti-inflammatory role of polyphenols. Nutrients.

[110] Kumar R., Caruso I.P., Ullah A., Cornelio M.L., Fossey M.A. (2017). Exploring the binding mechanism of flavonoid quercetin to phospholipase A2: Fluorescence spectroscopy and computational approach. Eur. J. Exp. Biol..

[111] Novo M., Hessel H., Fabri C., Ramos da Cruz Costa C., de Oliveira Toyama D., Domingues Passero L.F., Dalastra Laurenti M., Hikari Toyama M. (2017). Evaluation of rhamnetin as an inhibitor of the pharmacological effect of secretory phospholipase A2. Molecules.

[112] González Mosquera D.M., Hernández Ortega Y., Fernández P.L., González Y., Doens D., Vander Heyden Y., Pieters L. (2018). Flavonoids from Boldoa purpurascens inhibit proinflammatory cytokines (TNF-α and IL-6) and the expression of COX-2. Phytother. Res..

[113] Li Y., Yu Q., Zhao W., Zhang J., Liu W., Huang M., Zeng X. (2017). Oligomeric proanthocyanidins attenuate airway inflammation in asthma by inhibiting dendritic cells maturation. Mol. Immunol..

[114] Lin X., Lin C.H., Zhao T., Zuo D., Ye Z., Liu L., Lin M.T. (2017). Quercetin protects against heat stroke-induced myocardial injury in male rats: Antioxidative and anti-inflammatory mechanisms. Chemico-Biol. Interact..

[115] Galleggiante V., De Santis S., Cavalcanti E., Scarano A., De Benedictis M., Serino G., Chieppa M. (2017). Dendritic cells modulate iron homeostasis and inflammatory abilities following quercetin exposure. Curr. Pharm. Des..

[116] Gong J.H., Shin D., Han S.Y., Kim J.L., Kang Y.H. (2011). Kaempferol suppresses eosionphil infiltration and airway inflammation in airway epithelial cells and in mice with allergic asthma. J. Nutr..

[117] Weng Z., Patel A.B., Panagiotidou S., Theoharides T.C. (2015). The novel flavone tetramethoxyluteolin is a potent inhibitor of human mast cells. J. Allergy Clin. Immunol..

[118] Weng Z., Zhang B., Asadi S., Sismanopoulos N., Butcher A., Fu X., Theoharides T.C. (2012). Quercetin is more effective than cromolyn in blocking human mast cell cytokine release and inhibits contact dermatitis and photosensitivity in humans. PLoS ONE.

[119] Kim D.H., Jung W.S., Kim M.E., Lee H.W., Youn H.Y., Seon J.K., Lee J.S. (2014). Genistein inhibits pro-inflammatory cytokines in human mast cell activation through the inhibition of the ERK pathway. Int. J. Mol. Med..

[120] Veena S., Pracheta J. (2015). Protective assessment of *Euphorbia neriifolia* and its isolated flavonoid against N nitrosodiethylamine-induced Hepatic carcinogenesis in male mice: A histopathological analysis. Toxicol. Int..

[121] Lizhen Z., Chu W., Qiuxia M., Qin T., Yu N., Wei N. (2017). Phytochemicals of *Euphorbia lathyris* L. and their antioxidant activities. Molecules.

[122] Kerry N.L., Abbey M. (1997). Red wine and fractionated phenolic compounds prepared from red wine inhibits low density lipoprotein oxidation in vitro. Atherosclerosis.

[123] Hanan M., Ali S., Afaf E.A., Sayed A.E., Wagdi I.A., Wafaa H.B., Hanaa M. (2020). Nephroprotective and antioxidant activities of ethyl acetate fraction of *Euphorbia geniculata* Ortega family *Euphorbiaceae*. Arab. J. Chem..

[124] Claudia M., Furlan K., Pereira S., Martha D., Sedano-Partida L., Barbosa D., Deborah Y.A.C., Maria L., Giuseppina N., Paul E. (2015). Flavonoids and antioxidant potential of nine Argentinian species of Croton (Euphorbiaceae). Braz. J. Bot..

[125] Mishra B., Priyadarsini K., Kumar M., Unnikrishnan M., Mohan H. (2003). Effect of *O*-glycosilation on the antioxidant activity, free radical reactions of a plant flavonoid, chrysoeriol. Bioorg. Med. Chem..

[126] You H.J., Ahn H.J., Ji G.E. (2010). Transformation of rutin to antiproliferative quercetin-3-glucoside by *Aspergillus niger*. J. Agric. Food Chem..

[127] Juliana F., Jacyra A.S., Júlia M.F., Angela K.C., Yamara A.S.M., Elizabeth C.G., Denise V.T., Arnóbio A.S., Silvana M.Z., Matheus F. (2018). Comparison of two *Jatropha* species (*Euphorbiaceae*) used popularly to treat snakebites in Northeastern Brazil: Chemical profile, inhibitory activity against Bothrops erythromelas venom and antibacterial activity. J. Ethnopharmacol..

[128] Ajayi E.I.O., Adeleke M.A., Adewumi T.Y., Adeyemi A.A. (2017). Antiplasmodial activities of ethanol extracts of *Euphorbia hirta* whole plant and *Vernonia amygdalina* leaves in *Plasmodium berghei*-infected mice. J. Taibah Univ. Sci..

[129] Ashraf A., sarfraz R.A., Rashid M.A., Shahid M. (2015). Antioxidant, antimicrobial, antitumor, and cytotoxic activities of an important medicinal plant (*Euphorbia royleana*) from Pakistan. J. Food Drug Anal..

[130] Boumaza S., Bouchenak O., Yahiaoui K., Toubal S., El haddad D., Arab K. (2018). Effect of the aqueous extract of *Euphorbia guyoniana* (*euphorbiaceae*) on pathogenic bacteria from land-based sources. Appl. Ecol. Environ. Res..

[131] Beatriz G., Hélcio S., Paulo N., Bandeira T., Helena S., Rodrigues C., Matos F., Nascimento G.C., de Carvalho R., Braz-Filhoe M.R. (2020). Coutinhoa Evaluation of antibacterial and enhancement of antibiotic action by the flavonoid kaempferol 7-*O*-β-D-(6″-*O*-cumaroyl)-glucopyranoside isolated from *Croton piauhiensis müll*. Microb. Pathog..

[132] Ali R., Mat Houghton P.J., Raman A., Hoult J.R.S. (1998). Antimicrobial and anti-inflammatory activities of extracts and constituents of *Oroxylum indicum* (L.) Vent. Phytomedicine.

[133] Alcaráz L.E., Blanco S.E., Puig O.N., Tomás F., Ferretti F.H. (2000). Antibacterial activity of flavonoids against methicillin-resistant Staphylococcus aureus strains. J. Theor. Biol..

[134] Edziri H., Mastouri M., Mahjoub M.A., Mighri Z., Mahjoub A., Verschaeve L. (2012). Antibacterial, antifungal and cytotoxic activities of two flavonoids from *Retama raetam* flowers. Molecules.

[135] Jamil S. (2014). Antimicrobial Flavonoids from Artocarpus anisophyllus Miq and Artocarpus lowii King. J. Teknol..

[136] Farooq S., Wahab A.T., Fozing C., Rahman A.U., Choudhary M.I. (2014). Artonin I inhibits multidrug resistance in Staphylococcus aureus and potentiates the action of inactive antibiotics in vitro. J. Appl. Microbiol..

[137] Cushnie T.T., Lamb A.J. (2005). Detection of galangin-induced cytoplasmic membrane damage in Staphylococcus aureus by measuring potassium loss. J. Ethnopharmacol..

[138] Cushnie T.T., Lamb A.J. (2011). Recent advances in understanding the antibacterial properties of flavonoids. Int. J. Antimicrob. Agents.

[139] Ohemeng K.A., Schwender C.F., Fu K.P., Barrett J.F. (1993). DNA gyrase inhibitory and antibacterial activity of some flavones. Bioorg. Med. Chem. Lett..

[140] Xie Y., Yang W., Tang F., Chen X., Ren L. (2015). Antibacterial activities of flavonoids: Structure-activity relationship and mechanism. Curr. Med. Chem..

